# Enhancing the Performance of N‐Type Thermoelectric Devices via Tuning the Crystallinity of Small Molecule Semiconductors

**DOI:** 10.1002/advs.202204872

**Published:** 2022-11-27

**Authors:** Jiayao Duan, Jiamin Ding, Dongyang Wang, Xiuyuan Zhu, Junxin Chen, Genming Zhu, Chaoyue Chen, Yaping Yu, Hailiang Liao, Zhengke Li, Chong‐an Di, Wan Yue

**Affiliations:** ^1^ Guangzhou Key Laboratory of Flexible Electronic Materials and Wearable Devices School of Materials and Engineering Sun Yat‐Sen University Guangzhou 510275 P. R. China; ^2^ Beijing National Laboratory for Molecular Sciences CAS Key Laboratory of Organic Solids Institute of Chemistry Chinese Academy of Sciences Beijing 100190 P. R. China

**Keywords:** crystallinity tuning, n‐type small molecules, organic thermoelectric devices

## Abstract

In the development of high‐performance organic thermoelectric devices, n‐type materials, especially with small molecule semiconductors, lags far behind p‐type materials. In this paper, three small molecules are synthesized based on electron‐deficient naphthalene bis‐isatin building blocks bearing different alkyl chains with the terminal functionalized with 3‐ethylrhodanine unit and studied their aggregation and doping mechanism in detail. It is found that crystallinity plays an essential role in tuning the doping behavior of small molecules. Molecules with too strong crystallinity tend to aggregate with each other to form large crystalline domains, which cause significant performance degradation. While molecules with weak crystallinity can tolerate more dopants, most of them exhibit low mobility. By tuning the crystallinity carefully, organic thermoelectric devices based on **C12NR** can maintain high mobility and realize effective doping simultaneously, and a high power factor of 1.07 µW m^−1^ K^−2^ at 100 °C is realized. This delicate molecular design by modulating crystallinity provides a new avenue for realizing high‐performance organic thermoelectric devices.

## Graphical abstract

Through side chain engineering, three kinds of **NR** derivative small molecule semiconductors with different crystallinity are obtained for organic thermoelectric materials. By finely tuning the crystallinity, the thermoelectric devices based on **C12NR** maintain high mobility and effective chemical doping simultaneously and exhibit a high power factor of 1.07 µW m^−1^ K^−2^.

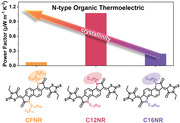

## 1 Introduction

Organic thermoelectric devices have attracted much attention for their applications in power generation for flexible electronic devices. Compared with the inorganic ones, organic thermoelectric materials are light‐weighted, flexible, and can be produced by solution process, and thus, considered as promising candidates applicable in thermoelectric devices.^[^ ^1^, ^2^, ^3^, ^4^ ^]^ The performance of thermoelectric devices is determined by the dimensionless figure of merit *zT* = *S* ^2^ *σT κ* ^−1^, where *S*, *σ*, *κ*, and *T* are the Seebeck coefficient, electrical conductivity, thermal conductivity, and temperature, respectively.^[^ ^5^, ^6^ ^]^ Since the thermal conductivity of organic thermoelectric materials is usually at a low level of <0.3 W mK^−1^, it is essential to improve the power factor (PF = *S* ^2^ *σ*) of materials by structure optimization. Up to date, many p‐type thermoelectric materials with high performances are reported,^[^ ^7^, ^8^, ^9^ ^]^ while less attention has been paid to the development of n‐type thermoelectric materials. However, to construct an efficient energy‐generating thermoelectric device, it is essential to utilize both high‐performance n‐type and p‐type thermoelectric materials to form complementary counterparts.^[^ ^10^ ^]^ To realize efficient electron transporting, highly electron‐deficient building blocks are commonly used to yield materials with low lowest unoccupied molecular orbital (LUMO).^[^ ^11^, ^12^, ^13^ ^]^ However, the synthesis and modification of materials with low LUMO are usually difficult due to the poor reactivity of highly electron‐deficient building blocks.^[^ ^14^, ^15^ ^]^

Up to date, most of the n‐type thermoelectric materials are based on conjugated polymers,^[^ ^16^, ^17^, ^18^, ^19^, ^20^ ^]^ but devices based on polymers usually suffer from batch‐to‐batch vibration, because the difference in molecular weight of polymers prepared in different batches can be significantly large. Compared with polymers, devices based on small molecules with definite molecular structures can avoid batch‐to‐batch vibration and possess excellent repeatability. Besides, the synthesis, modification, and purification of small molecules are relatively easier, making them promising candidates for organic thermoelectric devices.^[^ ^21^, ^22^, ^23^ ^]^ In the construction of n‐type organic semiconductors, much attention was paid to the strongly electron‐deficient naphthalene bis‐isatin (NB) building block for its application in the field of organic thermoelectric devices, organic field‐effect transistors, and organic electrochemical transistors.^[^ ^24^, ^25^, ^26^ ^]^ Materials constructed by naphthalene bis‐isatin building blocks are linked by a double bond, which can form a rigid backbone and avoid the rotation between adjacent aromatic rings. Besides, the strong electron‐withdrawing lactam group in naphthalene bis‐isatin effectively lowers the LUMO level and facilitates electron transport, showing impressive air‐stable, high‐mobility electron transport in top‐gate OTFTs.^[^ ^27^, ^28^ ^]^ In 2020, I. McCulloch and co‐workers reported the synthesis of copolymers constructed by naphthalene bis‐isatin building blocks, and the resulting thermoelectric devices can achieve a power factor of 0.16 µW m^−1^ K^−2^.^[^ ^29^ ^]^ In the following works, the power factor was enhanced to 3.2 µW m^−1^ K^−2^ by optimization of the backbone structure,^[^ ^30^ ^]^ and further pushed to 10.4 µW m^−1^ K^−2^ by side‐chain engineering.^[^ ^31^ ^]^ From these reports, we can conclude that naphthalene bis‐isatin is a promising building block for high‐performance organic thermoelectric materials. However, to the best of our knowledge, there is no report of small molecules based on naphthalene bis‐isatin building blocks for organic thermoelectric devices.

In the development of the aforementioned thermoelectric materials, doping of the active layer is a widely used strategy to enhance the device's performance.^[^ ^32^, ^33^ ^]^ To form an ideal doping film for high‐performance devices, the aggregation and crystallinity of materials play an important role in the doping process.^[^ ^34^ ^]^ However, there are seldom studies to investigate the relationship between the crystallinity and device performance of materials. It is believed that a low tendency of aggregation can enhance the miscibility of organic semiconductors with dopants, and suppress phase separation. However, the enhanced miscibility will weaken the crystallinity of organic semiconductors, which results in lower charge mobility, and thus, lower power factors of devices.^[^ ^35^, ^36^, ^37^, ^38^, ^39^ ^]^ It is challenging to balance the miscibility and crystallinity of organic semiconductors for the development of high‐performance organic thermoelectric devices.

It is widely believed that side chain engineering is an efficient strategy to tune the crystallinity of organic semiconductors, and the study of properties and device performance of molecules with different side chains can provide us with a clear relationship between chemical structure, crystallinity, and device performance of organic thermoelectric materials. Usually, a molecule with a shorter side chain is of stronger crystallinity,^[^ ^40^ ^]^ and fluorination of the side chain can enhance the crystallinity dramatically.^[^ ^41^, ^42^ ^]^ In this paper, three novel fused n‐type small molecules with the same backbone but different side chains were designed for thermoelectric devices. By side chain engineering, the crystallinity of these molecules was fully optimized. We found that longer alkyl chains hurt the molecular packing and result in a weaker crystallinity and charge transport ability, and partially fluorinated alkyl chains bring the molecules with much more crystalline domains, but the much stronger aggregation tendency also makes them difficult to blend with dopants. Finally, **C12NR** with shorter alkyl chains was obtained to form ideal doping with N‐DMBI, and in the meantime, high crystallinity was maintained. As a result, the devices based on **C12NR** exhibit the highest electron mobility of 1.29 S cm^−1^, as well as the highest power factor of 0.69 µW m^−1^ K^−2^ at room temperature and 1.07 µW m^−1^ K^−2^ at 100 °C. The relationship between crystallinity and device performance investigated in this paper can provide significant referential importance for the design of high‐performance n‐type thermoelectric materials.

## 2 Results and Discussion

**Figure 1:**
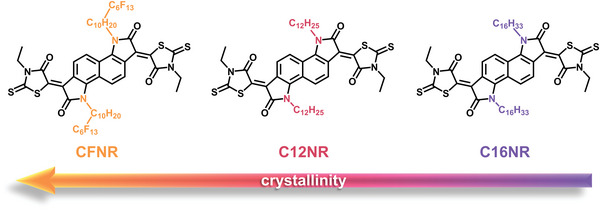
The molecular structures of **CFNR**, **C12NR**, and **C16NR**.

The chemical structures are shown in **Figure** **1**. All three molecules have the same backbone structure of fused rings constructed by Knoevenagel condensation of naphthalene bis‐isatin monomer and 3‐ethylrhodanine with a high yield,^[^ ^26^ ^]^ and the solubility of the resulting three molecules is quite good and can be soluble in common used organic solvents such as chloroform and toluene. Although with the same backbone structure, their side chains are quite different from each other. **C12NR** has the shortest side chain of C_12_H_25_, while **C16NR** has a longer side chain of C_16_H_33_. The length of the side chain in **CFNR** is the same as **C16NR**, but six alkyl groups of the side chain are fluorinated, resulting in a structure of C_10_H_20_C_6_F_13_. All the synthetic details can also be seen in the Supporting Information, and all the chemical structures were fully characterized by proton and carbon nuclear magnetic resonance (^1^H NMR and ^13^C NMR) spectroscopy and matrix‐assisted laser desorption–ionization time of flight (MALDI‐TOF) mass spectrometry. From the thermogravimetric analysis (TGA) (Figure S1, Supporting Information), all the **NR** derivatives show good thermal stabilities with a decomposition temperature of 358 °C for **CFNR**, 356 °C for **C12NR**, and 352 °C for **C16NR**, respectively. As determined by the differential scanning calorimetry (DSC) analysis (Figure S2, Supporting Information), **CFNR** presents a melting temperature (*T*_m_) of 324 °C and crystallization temperature (*T*_c_) of 299 °C, **C12NR** presents a *T*_m_ of 294 °C and *T*_c_ of 288 °C, and **C16NR** presents a *T*_m_ of 275 °C and *T*_c_ of 263 °C. From the decreasing melting temperature and crystallization temperature from **CFNR**, **C12NR**, to **C16NR**, it is reasonable to infer that the crystallinity of **CFNR** is stronger than **C12NR**, and **C16NR** has the weakest crystallinity.^[^ ^43^ ^]^ In addition, the molar melting enthalpy (∆*H*_m_) and molar crystallization enthalpy (∆*H*_c_) were calculated for further interpreting the crystallinity.^[^ ^44^ ^]^ **CFNR** exhibits the highest ∆*H*_m_ of 40.18 kJ mol^−1^ and ∆*H*_c_ of 25.14 kJ mol^−1^, followed by **C12NR** with the ∆*H*_m_ of 38.87 kJ mol^−1^ and ∆*H*_c_ of 14.30 kJ mol^−1^, and **C16NR** exhibits the lowest ∆*H*_m_ of 34.52 kJ mol^−1^ and ∆*H*_c_ of 12.73 kJ mol^−1^. The decreasing of molar melting enthalpy and molar crystallization enthalpy from **CFNR**, **C12NR**, to **C16NR** are well consistent with the crystallinity discussed above.

First, we studied the optical and electrical properties of **CFNR**, **C12NR**, and **C16NR** by UV–vis–near‐infrared (NIR) spectra and cyclic voltammetry (CV) measurements (Figure S3, Supporting Information). The detailed data are summarized in **Table** **1**. Generally, thin films spin‐coated from the three small molecule semiconductors exhibit a similar absorption spectrum with dual‐band absorption bands, corresponding to the glycolated **NR** derivatives from the previous research.^[^ ^26^ ^]^ One is the high‐energy band originating from strong *π*–π* characters with an absorption onset of around 500 nm, and the other one is the intramolecular charge transfer (ICT) absorption with an absorption onset of around 1000 nm, by which we can estimate the bandgap of them to be 1.36 eV for **C12NR** and 1.35 eV for both **CFNR** and **C16NR**. The difference is too small to identify, meaning that side‐chain engineering makes a tiny difference in the bandgaps of these molecules. However, the absorption spectra of **CFNR** show a significant red‐shifted from the solution to the film state, which can be attributed to the strong aggregation of fluorinated alkyl chains.^[^ ^45^, ^46^, ^47^ ^]^ By CV, the detailed HOMO/LUMO energy level of **CFNR**, **C12NR**, and **C16NR** was determined to be −3.95/−5.30 eV, −3.90/−5.26 eV, and −3.95/−5.30 eV. Attribute to the fused electron‐withdrawing groups, the LUMO of all **NR** derivatives is very close to −4 eV, which is believed to be essential to facilitate the highly efficient electron transporting of materials in devices. From the corresponding optical bandgaps and LUMO levels, we can calculate the highest occupied molecular orbital (HOMO) to be −5.26 eV for **C12NR** and −5.30 eV for both **CFNR** and **C16NR**. Considering the measurement error of CV, we can draw a conclusion that the influence of side chains on energy level and band gaps is negligible.

**Table 1:** Summary of optoelectronic properties for **CFNR**, **C12NR**, and **C16NR**

Compound	*λ*_ICT_^abs(solution)^ [nm]^a)^	*λ*_ICT_^abs(film)^ [nm]^b)^	*λ*_onset_^abs^ [nm]^c)^	*E*_g_^opt^ [eV]^d)^	*E*_1r_ [V]^e)^	*E*_LUMO_ [eV]^f)^	*E*_HOMO_ [eV]^g)^
**CFNR**	688	704	916	1.35	−0.85	−3.95	−5.30
**C12NR**	687	688	909	1.36	−0.90	−3.90	−5.26
**C16NR**	688	690	921	1.35	−0.85	−3.95	−5.30

^a)^ Maximum ICT absorption peak in solution;

^b)^ Maximum ICT absorption peak in thin films;

^c)^ The onset of film absorption ;

^d)^ Optical band gap calculated by *E*_g_^opt^ = 1240/*λ*_onset_^abs^;

^e)^ The onset of the first reduction wave in a 0.1 м tetrabutylammonium hexafluorophosphate in dichloromethane solution (*E*_1r_ vs Fc/Fc+);

^f)^ Estimated from the onset of the first reduction wave using the equation, *E*_LUMO_ = −(4.80 + *E*_1r_);

^g)^ Calculated from *E*_HOMO_ = E_LUMO_ − *E*_g_^opt^.

**Figure 2:**
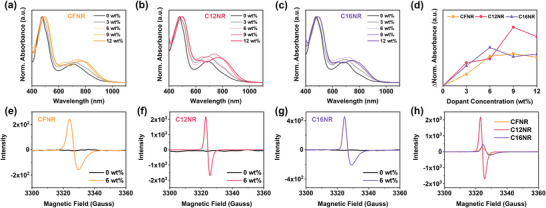
Normalized UV–vis–NIR absorption spectra of a) **CFNR**, b) **C12NR**, and c) **C16NR** in thin films; d) Absolute change of polaron absorption under different dopant concentrations of N‐DMBI; X‐band ESR spectra of e) **CFNR**, f) **C12NR**, and g) **C16NR** before and after introduction of N‐DMBI in solution; h) X‐band ESR spectra of **CFNR**, **C12NR**, and **C16NR** after introduction of N‐DMBI in solution.

To investigate the interaction of dopant with the three novel small molecules semiconductors, the UV–vis–NIR absorption spectra of **CFNR**, **C12NR**, and **C16NR** mixing with a dopant in a different ratio ranging from 0 to 12 wt% were also measured in both solutions (Figure S4, Supporting Information) and films (**Figure** **2**a–d). When the dopant ratio increases from 0 to 12 wt%, we can find that the intensity of *π*–π* and ICT absorption is decreased and a new absorbance peak at 820 nm appears, which implies charge transfer from N‐DMBI to an organic semiconductor.^[^ ^48^, ^49^ ^]^ The UV–vis–NIR spectra of all three semiconductors mixing with a dopant in chloroform solution exhibit the same trend, indicating that all three molecules make no difference in the formation of the molecular complex when mixed with a dopant in solution. This is because the good solubility of these three molecules makes it possible for the molecules and dopants to move freely until the molecular complex forms. The UV–vis–NIR absorption spectra of **CFNR**, **C12NR**, and **C16NR** mixing with a dopant in a different ratio ranging from 0 to 12 wt% in films were also measured. From Figure 2a, we can find that as the ratio of dopant increases from 0 to 12 wt%, the peak of **CFNR** at 706 nm became weak, and two new peaks emerge at 636 and 765 nm, which is usually attributed to the formation of polarons.^[^ ^50^ ^]^ The trend of UV–vis–NIR absorption spectra of **C12NR** and **C16NR** is the same as **CFNR**. As the increasing of dopant ratio, two new peaks appear at 633/763 nm and 639/743 nm for **C12NR** and **C16NR**, respectively. Besides, the intensity of the new peak at 633/763 nm **C12NR** is much higher than that of **CFNR** and **C16NR** (Figure 2d), and this reveals that the chemical doping of **C12NR** is more effective than that of **CFNR** and **C16NR**.^[^ ^51^ ^]^

**Figure 3:**
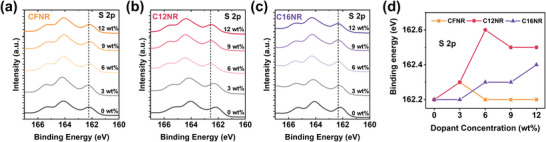
S (2p) XPS spectra of a) **CFNR**, b) **C12NR**, and c) **C16NR** thin film under different dopant concentrations of N‐DMBI; d) Absolute shift of binding energies of S (2p) under different dopant concentrations of N‐DMBI.

To further understand the difference in doping of these three molecules, electron spin resonance (ESR) spectroscopy and X‐ray photoelectron spectroscopy (XPS) spectra characterization were conducted. From the ESR spectra in Figure 2e–h, we can find that all three small molecules exhibit no signal in solutions without a dopant, and strong radical anion peaks appear at *g* = 1.9770, 1.9786, and 1.9770 for **CFNR**, **C12NR**, and **C16NR**, respectively, indicating strong charge transfer between small molecules and dopants occurs in the solution with 6 wt% N‐DMBI. It is worth noting that the radical anion peak of **C12NR** is much stronger than that of **CFNR** and **C16NR**, by which we can draw the conclusion that the chemical doping of **C12NR** is the most efficient in solution states. The XPS results of the pristine and doped films are shown in **Figure** **3** and Figure S5, Supporting Information. The binding energy of O 1s, N 1s, and S 2p of **C16NR** shifts from 531.7, 400.2, and 162.2 eV for the pristine film to 532, 400.4, and 162.3 eV for doped films. This shift is usually attributed to the n‐doping‐induced electron transfer from dopant to **C16NR**. For **C12NR**, the binding energy of O 1s, N 1s, and S 2p also shift from 531.3, 400.2, and 162.2 eV for the pristine film to 531.8, 400.4, and 162.6 eV for doped films. For **CFNR**, the binding energy of O 1s, N 1s, and S 2p also shift from 400.2, 531.3, and 162.2 eV for the pristine film to 400.4, 531.5, and 162.3 eV for doped films. In general, **C12NR** shows the most obvious shift of binding energies which especially in the S 2p spectrum (Figure 3d), indicative of a strong charge transfer for N‐DMBI‐doped **C12NR**, and this is consistent with the conclusion we draw from the ESR spectra and UV–vis–NIR absorption spectra.

**Figure 4:**
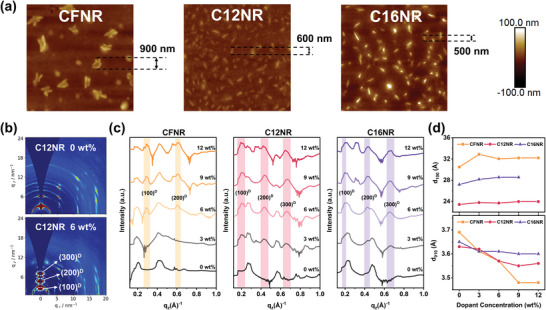
a) AFM images (10 × 10 µm) of semiconductor films of **CFNR**, **C12NR**, and **C16NR** with N‐DMBI. b) 2D‐GIWAXS images of **C12NR** thin film with 0 and 6 wt% N‐DMBI; c) Line‐cut profiles of **CFNR**, **C12NR**, and **C16NR** films obtained by integration along the out‐of‐plane (*q_z_*) direction; d) Absolute shift of d‐spacing of d_100_ at out‐of‐plane (*q_z_*) direction and d_010_ at in‐plane (*q_xy_*) direction under different dopant concentrations of N‐DMBI.

The morphology of blending films of **CFNR**, **C12NR**, and **C16NR** with different ratios of dopant is also studied by atomic force microscope (AFM) experiment. From the AFM image in **Figure** **4**a and Figure S6, Supporting Information, we can find that these three molecules exhibit different blending behaviors. For **C16NR** with the longest alkyl chain, a uniform film can be formed when 0, 3, and 6 wt% dopants are added, and the dopant tend to crystallize and small domains of dopant can be observed when 9 wt% dopant is added, with a domain size of 500 nm (9 wt%). For **C12NR**, as the increasing of dopant ratio, dopant domains appear when 6 wt% dopant is added, and the domain size is 600 nm. But for **CFNR** with the fluorinated alkyl chains, large domains with a domain size of 900 nm emerge clearly in the films with ≤6 wt% dopant added, this is because the high crystallinity of **CFNR** makes it difficult to mix well with a dopant, leaving the dopant to form pure domains. From the length and fluorination of the side chain, we can estimate that the crystallinity increases for **C16NR**, **C12NR**, and **CFNR**, so it is clear that the tolerance of dopant for these three molecules is decreasing as the crystallinity increases.

To further study the detailed difference of intermolecular stacking in films between these three molecules, 2D grazing incident wide‐angle X‐ray scattering (GIWAXS) experiment was conducted. The 2D GIWAXS images are provided in Figure 4b–d and Figure S7, Supporting Information, and the detailed data are summarized in Tables S1–S3, Supporting Information. All three molecules exhibit strong lamellar stacking in pristine films, with the 100 and 200 peaks being identified clearly in the 2D GIWAXS image. The lamellar stacking distances of **C16NR**, **C12NR**, and **CFNR** from the pristine films are 27.2, 23.4, and 30.5 Å, respectively, consistent with the length of their side chains. For **C12NR** in Figure 4b, peaks at 0.202 (100^D^), 0.430 (200^D^), and 0.696 Å^−1^ (300^D^) appear when 3 wt% dopant is added to the films, and these peaks become stronger when the dopant ratio is 6 wt%, which might be induced by changing of packing behaviors of **C12NR** when mixed with dopants. With further increase of dopant ratio of 9 and 12 wt%, the out‐of‐plane profile of **C12NR** is dominated by 100^D^, 200^D^, and 300^D^ peaks. The obvious shrinks of origin 100 and 200 peaks are probably due to the destruction from dopant to **C12NR** in molecular packing. The same phenomenon can be found in **C16NR**, while for **CFNR**, two peaks appear at 0.289 (100^D^) and 0.603 Å^−1^ (200^D^) when the dopant ratio is 6 wt%, indicatives of difficult doping. Due to the excessive phase separation between the molecule and dopant, intermolecular stacking of **CFNR** is thus not influenced by the intruding of the dopant. Compared with their out‐of‐plane 010 peaks, all three small molecules show much clearer in‐plane 010 peak in films, which is corresponding to the preference for edge‐on stacking orientation. For the pristine films, three molecules exhibit comparable *π*–*π* stacking distance of 3.65 Å for **C16NR**, 3.63 Å for **C12NR**, and 3.68Å for **CFNR**, respectively. However, **C12NR** and **C16NR** exhibit edge‐on orientation with dot‐like scattering diffraction, on the contrary, **CFNR** shows obvious edge‐on orientation with concentrated scattering diffraction, which corresponds to the highest crystallinity of **CFNR**. As the ratio of dopant increases from 0 to 12 wt%, the out‐of‐plane lamellar stacking distance becomes larger, but the in‐plane *π*–*π* stacking distance becomes smaller. For **CFNR,** its in‐plane *π*–*π* stacking distance changed from 3.68 (0 wt% dopant) to 3.48 Å (12 wt% dopant), which should be attributed to the enhanced self‐aggregation induced by severe phase separation when it is mixed with a dopant, and for **C16NR**, its long alkyl chain makes it possible to blend well with a dopant, so it has the weakest self‐aggregation, which result in a slight change of in‐plane *π*–*π* stacking distance from 3.65 (0 wt% dopant) to 3.60 Å (12 wt% dopant). The change of in‐plane *π*–*π* stacking distance for **C12NR** is between **CFNR** and **C16NR**, indicating it can maintain both a relatively weak self‐aggregation and an orderly *π*–*π* stacking, and this molecular packing is beneficial for the device performance.^[^ ^52^ ^]^

**Figure 5:**
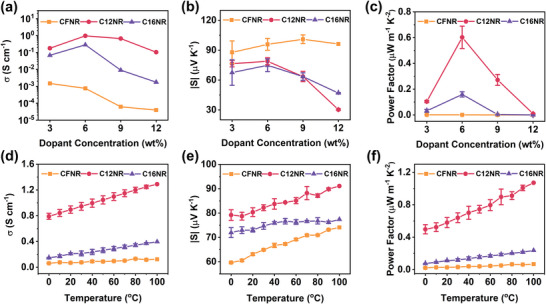
Thermoelectric properties of **CFNR**, **C12NR**, and **C16NR** doped with N‐DMBI. a) Electrical conductivities, b) Seebeck coefficients, and c) power factors under different dopant concentrations of N‐DMBI. Temperature dependence of d) electric conductivity, e) Seebeck coefficient, and f) power factor of 3 wt% N‐DMBI‐doped **CFNR**, 3 wt% N‐DMBI‐doped **C12NR**, and 6 wt% N‐DMBI‐doped **C16NR**.

The thermoelectric devices of **C16NR**, **C12NR**, and **CFNR** were prepared and the electrical conductivity and Seebeck coefficient were measured to evaluate the device performance (**Figure** **5** and **Table** **2**). For **C16NR**, as the dopant ratio increased, a peak electrical conductivity of 0.29 S cm^−1^ appears when 6 wt% dopant is added. Further increase of dopant ratio to 9 wt% results in a dramatic decrease of electrical conductivity to 0.009 S cm^−1^. For **C12NR**, the trend of dependence of electrical conductivity on dopant ratio is the same as **C16NR**, with a maximum electrical conductivity of 0.98 S cm^−1^ for 6 wt% doped film. Unlike **C16NR** and **C12NR**, the maximum electrical conductivity (1.5 × 10^−3^ S cm^−1^) of **CFNR** appears when 3 wt% dopant is added, and further increase of dopant ratio leads to a decrease of electrical conductivity to 6 × 10^−5^ S cm^−1^ (9 wt% dopant). Generally, the electrical conductivity of **CFNR** is much lower than that of **C16NR** and **C12NR**, and this can be attributed to its poor chemical doping ability. As for the Seebeck coefficient, the maximum Seebeck coefficient is −79 µV K^−1^ for **C12NR** and −75 µV K^−1^ for **C16NR** both with 6 wt% N‐DMBI. As for **CFNR**, the Seebeck coefficient increase from −88 to −101 µV K^−1^ when the dopant ratio increases from 3 to 9 wt%. From the electrical conductivity and Seebeck coefficient, we can calculate the PFs of devices based on the three molecules. Of the three molecules, **C12NR** has the highest PFs of 0.69 µW m^−1^ K^−2^ when 6 wt% dopant is used, and an increase or decrease of dopant ratio will hurt the device performance to a low value. For **C16NR**, the peak PFs also appears when 6 wt% dopant is used, but the value (0.18 µW m^−1^ K^−2^) is relatively lower than that of **C12NR**. As for **CFNR**, the maximum PFs of 8.2 × 10^−4^ µW m^−1^ K^−2^ at 3 wt% dopant used is much lower than that of **C12NR**. The device performance of **C16NR**, **C12NR**, and **CFNR** under different temperatures is also investigated. As the temperature increase from 20 to 100 °C, both the electrical conductivity and Seebeck coefficient of **C12NR** film are increased from 0.89 to 1.29 S cm^−1^, and −80 to −91 µV K^−1^, respectively, resulting in an increase of PFs from 0.58 to 1.07 µW m^−1^ K^−2^. The trend of **C16NR** and **CFNR** films is the same as **C12NR**. From 20 to 100 °C, the overall PFs increased from 0.11 to 0.24, and 0.024 to 0.066 µW m^−1^ K^−2^, respectively. Hall effect measurements were carried out to study the carrier concentration and mobility of doped films. **C12NR** shows Hall mobility of 0.002 cm^2^ V^−1^ s^−1^ and carrier concentration of 5.63 × 10^20^ cm^−3^ with 6 wt% N‐DMBI, while **CFNR** and **C16NR** are failed to measure the Hall signal due to poor electricity, which indicates that the crystallinity of the small molecule semiconductors needs to be optimized delicately to improve the devices’ performance.

**Table 2:** Electric conductivity, Seebeck coefficients, and power factor values of N‐DMBI‐Doped **CFNR**, **C12NR**, and **C16NR** films

Compound	*T*	*σ* [S cm^−1^]	*S* [µV K^−1^]	PF [µW m^−1^ K^−2^]
**CFNR**	r.t.^a)^	1.5 × 10^−4^	−88 ± 11	(7 ± 1.2) × 10^−4^
	353 K	0.13 ± 0.01	−71 ± 0.2	(6.6 ± 0.2) × 10^−2^
**C12NR**	r.t.	0.97 ± 0.1	−79 ± 4	0.60 ± 0.09
	373 K	1.29	−91	1.07
**C16NR**	r.t.	0.29 ± 0.02	−75 ± 6	0.16 ± 0.02
	373 K	0.40	−77	0.24

^a)^ r.t. represents room temperature.

**Figure 6:**
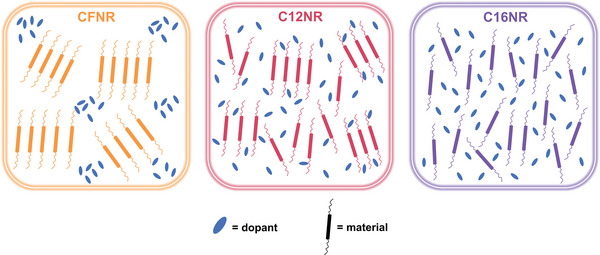
Schematic of blending mechanism of **C16NR**, **C12NR**, and **CFNR** with dopant.

The blending mechanism of **C16NR**, **C12NR**, and **CFNR** with dopant can be proposed as **Figure** **6** shows. In general, the crystallinity of small molecules dominated the aggregation behavior in the formation of films. Due to the good solubility of the small molecules, all of them can mix well with the dopant in the solution. As the solvent is removed in the process of film formation, **CFNR** with fluorinated alkyl chain and strongest crystallinity tends to aggregate with other **CFNR** molecules, resulting in large and high crystalline **CFNR** domains, and the dopant (even with a low percentage) is left to form pure dopant domains. The strong self‐aggregation of **CFNR** results in films with the strongest phase separation and most crystalline domains, but it also brings with least effective doping, which is disadvantageous for the device's performance. When **CFNR** is replaced by **C16NR** with non‐fluorinated alkyl chains, the weakest crystallinity of these three molecules makes **C16NR** exhibit the lowest self‐aggregate tendency, so it can only form the smallest domains and tolerant most dopant in the process of film formation. This means the doping of **C16NR** is the most effective, but the highest miscibility with dopant destroys the molecular stacking and hurts the charge transport of **C16NR**, which is also not beneficial for the device's performance. For **C12NR,** it has a shorter and non‐fluorinated alkyl chain, so its crystallinity and self‐aggregation tendency is between **CFNR** and **C16NR**. The moderate self‐aggregation tendency of **C12NR** makes it possible to form high crystalline but not so large domains of **C12NR,** and at the same time, the dopant is allowed to crystallize between these **C12NR** domains, and thus, effective doping is formed in uniform films. In summary, balancing between self‐aggregation and effective doping can be realized by side‐chain engineering to finely tune the crystallinity of small molecules.

## 3 Conclusion

In conclusion, three n‐type small molecules with different side alkyl chains (**C16NR**, **C12NR**, and **CFNR**) are synthesized for organic thermoelectric devices. UV–vis–NIR spectra and XPS indicate effective chemical doping when these small molecules are mixed with dopants both in solution and films and by AFM and 2D‐GIWAXS experiments, the aggregation and doping mechanisms are studied in detail. **C16NR** with the longest alkyl chains are of the weakest crystallinity and self‐aggregation tendency, so it can mix well with a dopant in the films and very little amount of dopant is left to form pure dopant crystals. For **CFNR**, the fluorinated alkyl chain brings it the strongest crystallinity, but its strongest self‐aggregation tendency also brings it the poorest miscibility with dopants. Compared with **C16NR** and **CFNR**, the shorter alkyl chain of **C12NR** facilitates it with compact molecular stacking. In the meantime, it can blend well with the dopant and can form excellent chemical doping. As a result of maintaining good crystallinity and film morphology, a high PF of 1.07 µW m^−1^ K^−2^ is obtained for the thermoelectric device based on **C12NR**. Based on the experimental results, we can draw the conclusion that crystallinity plays an essential role in tuning the doping behavior of small molecules. To realize devices with high performance, the crystallinity needs to be tuned carefully to obtain films with high mobility and effective doping with a dopant.

## 4 Experimental Section

### Materials

All starting materials were purchased from commercial suppliers and used as received unless otherwise specified. 1,5‐naphthalenediamine was purchased from Bide Medical (Shanghai) Co., Ltd. 3‐ethylrhodanine was purchased from Macklin Medical Co., Ltd. The solvents purchased from Sigma Aldrich for spectroscopic studies were of spectroscopic grade and used as received. The intermediates and target small molecules were purified by column chromatography on silica gel (General‐Regent, 200–300 mesh).

### Material Structures, Optoelectronic Properties, and Morphologies Characterization

^1^H and ^13^C NMR spectra were recorded in CDCl_3_ with a 400 MHz Bruker Advance III spectrometer. UV–vis–NIR absorption spectra were recorded on a UV‐1601 Shimadzu UV–vis–NIR spectrometer. The small molecular films were spin‐coated on glass substrates from chlorobenzene/chloroform = 7/3 v/v solution (3 mg mL^−1^) under ambient conditions. TGA was carried out on a Thermogravimetric Analyzer from Nicolet 6700 at a rate of 10 °C min^−1^ under a nitrogen atmosphere. DSC experiments were carried out with a Netzsch DSC‐204 F1 instrument at a heating rate of 10 °C min^−1^ under nitrogen. Mass spectra were recorded on an AB Sciex‐5800 MALDI‐TOF mass spectrometer and a Bruker Solarix XR mass spectrometer. CV was performed on a standard commercial electrochemical analyzer (Shanghai Chenhua Instrument co. LTD., CHI520E) with a three‐electrode system consisting of a cylindrical platinum working electrode, platinum wire counter electrode, and Ag/AgCl reference electrode. The potential of the Ag/AgCl reference electrode was internally calibrated against ferrocene. 0.1 m tetrabutylammonium hexafluorophosphate (TBAPF_6_) in deoxygenated dichloromethane was used as the supporting electrolyte.

### X‐ray Photoelectron Spectroscopy

The XPS measurements were carried out in a SHIMADZU AXIS SUPRA+ ultra‐high‐vacuum (5 × 10^−10^ Torr) photoelectron spectroscopy with a monochromatic Al K*α* X‐ray (1486.6 eV) excitation source. All the samples were fabricated on pure silicon substrates in an N_2_ atmosphere.

### Measurement of Electrical Conductivity

The gold electrodes on a glass substrate were patterned by photolithography with a channel length and width of 500 and 1000 µm, respectively. After the substrates were cleaned by distilled water, ethanol, and acetone, successively, the doped films were drop‐coated on the glass substrates and annealed at 120 °C for 8 h. Thereafter, Cytop was spin‐coated on the surface of the films to serve as an encapsulation layer. The four‐probe method was used to measure the electrical conductivity with an Agilent B1500A. All the preparations and measurements were carried out in a glove box under a nitrogen atmosphere.

### Measurement of Seebeck Coefficient

The measurement of the Seebeck coefficient was performed in a vacuum chamber. The temperature gradient was created by using two Peltier modules. The built temperature differences were measured by a FLIR A300, an infrared camera with a temperature sensitivity of less than 50 mK. Seebeck coefficient can be calculated by *S* = Δ*V*/Δ*T*, where Δ*V* is the thermal voltage between the two electrodes of the device when applied a temperature difference of Δ*T*. The Δ*V* was measured by Agilent B1500A in a vacuum.

### AFM Measurements

AFM measurements were performed in tapping mode, using Bruker dimension icon AFM with a silicon tip. The small molecular films were spin‐coated on glass substrates from chlorobenzene/chloroform = 7/3 v/v solution (3 mg mL^−1^) under ambient conditions.

### GIWAXS Characterization

Samples for X‐ray scattering were prepared using the same procedures for the preparation of active channel layer in device fabrication except using pure silicon wafers as the substrates. An incidence angle of 0.18° and photon energy of 8 keV were used to record the scattering patterns. The 2D GIWAXS patterns were collected from films with a resistivity of 0.001–30 Ohm cm^−1^.

## Conflict of Interest

The authors declare no conflict of interest.

## Supporting information

Supporting Information

## Data Availability

The data that support the findings of this study are available from the corresponding author upon reasonable request.
