# Supplementary material for: Enhancing the Performance of N‐Type Thermoelectric Devices via Tuning the Crystallinity of Small Molecule Semiconductors
Source: Adv Sci (Weinh). 2022 Nov 27;10(3):2204872. doi: 10.1002/advs.202204872 (PMC9875661; doi:10.1002/advs.202204872)
Supplement: Supplementary file 1 — Supporting Information [file ADVS-10-2204872-s001.pdf]

ADVANCED SCIENCE
Open Access

Supporting Information

for *Adv. Sci.*, DOI 10.1002/advs.202204872

Enhancing the Performance of N-Type Thermoelectric Devices via Tuning the Crystallinity of Small Molecule Semiconductors

Jiayao Duan, Jiamin Ding, Dongyang Wang, Xiuyuan Zhu, Junxin Chen, Genming Zhu, Chaoyue Chen, Yaping Yu, Hailiang Liao, Zhengke Li, Chong-an Di* and Wan Yue**

Supporting Information

Enhancing the performance of n-type thermoelectric devices via tuning the crystallinity of small molecules semiconductors

Jiayao Duan‡, Jiamin Ding‡, Dongyang Wang, Xiuyuan Zhu, Junxin Chen, Genming Zhu, Chaoyue Chen, Yaping Yu, Hailiang Liao, Zhengke Li, Chong-an Di*, Wan Yue**

Table of Contents

1. **TGA of Compounds**
2. **DSC of Compounds**
3. **CVs of Compounds**
4. **UV-vis-NIR of Compounds**
5. **XPS spectra**
6. **AFM Images of Compounds**
7. **The Details of the GIWAXs Study**
8. **Hall effect measurements**

9. Material Synthesis and Characterization

10. References

1. TGA of Compounds

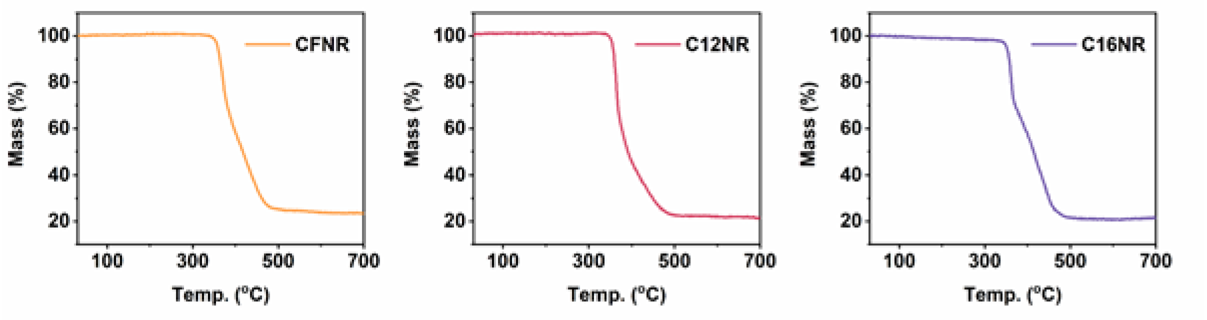

Figure S1. TGA of **CFNR**, **C12NR** and **C16NR** measured in nitrogen.

2. DSC of Compounds

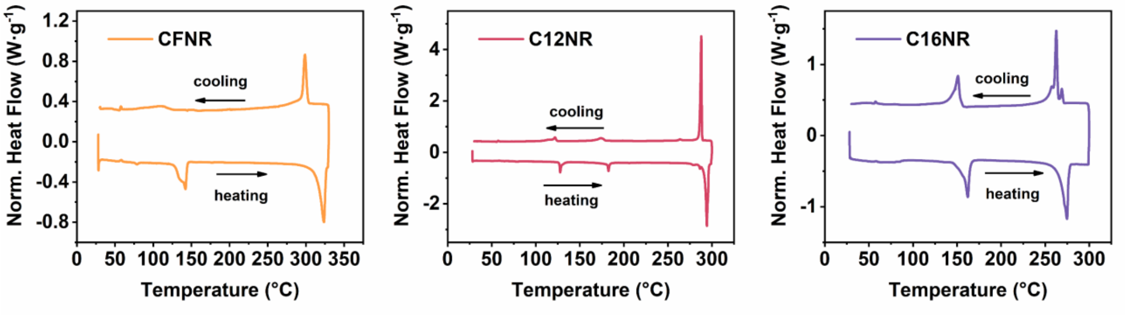

Figure S2. DSC of **CFNR**, **C12NR** and **C16NR** measured in nitrogen.

3. CVs of Compounds

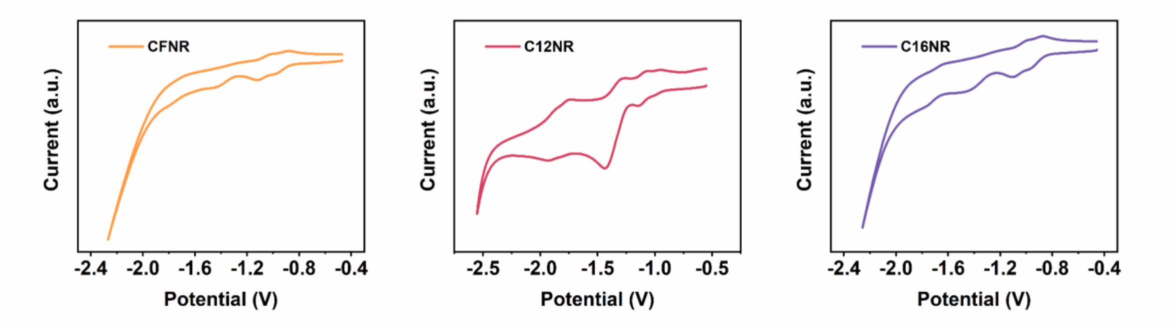

Figure S3. Cyclic voltammetry curves of **CFNR**, **C12NR** and **C16NR** in dichloromethane solution with 0.1 M n-Bu_4NPF_6 as supporting electrolyte at a scan rate of 0.1 V/s. The potential of Ag/AgCl reference electrode was internally calibrated against ferrocene.

4. UV-vis-NIR of Compounds

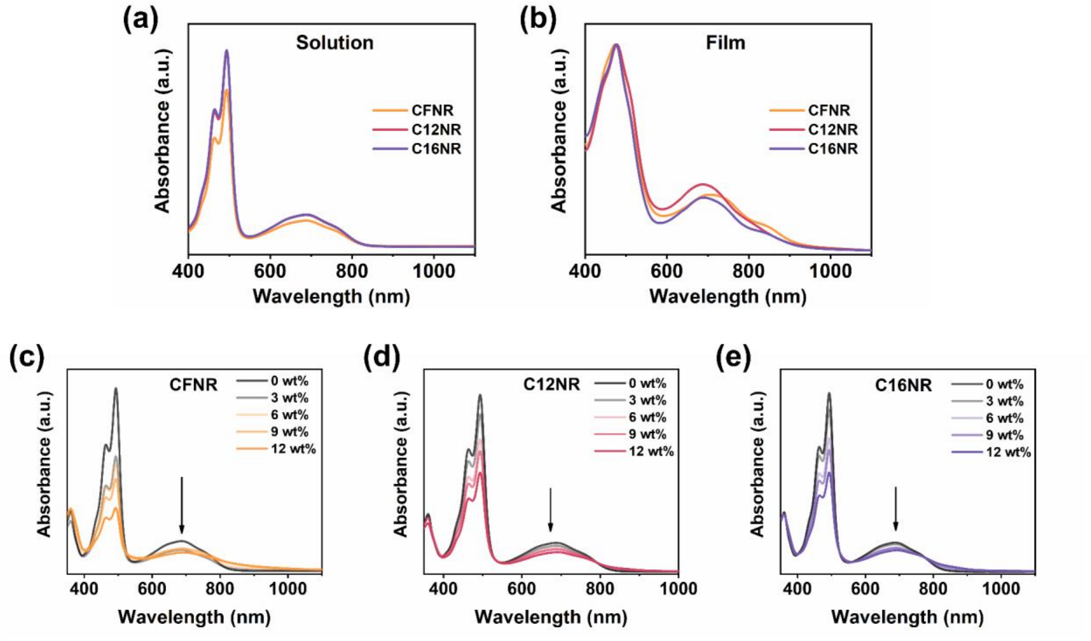

Figure S4. (a) UV-vis-NIR absorption spectra of **CFNR**, **C12NR** and **C16NR** in chloroform solution; (b) Normalized UV-vis-NIR absorption spectra of **CFNR**, **C12NR** and **C16NR** in thin films; UV-vis-NIR absorption spectra of (c) **CFNR**, (d) **C12NR** and (e) **C16NR** in chloroform solution under different dopant concentration of N-DMBI.

5. XPS spectra

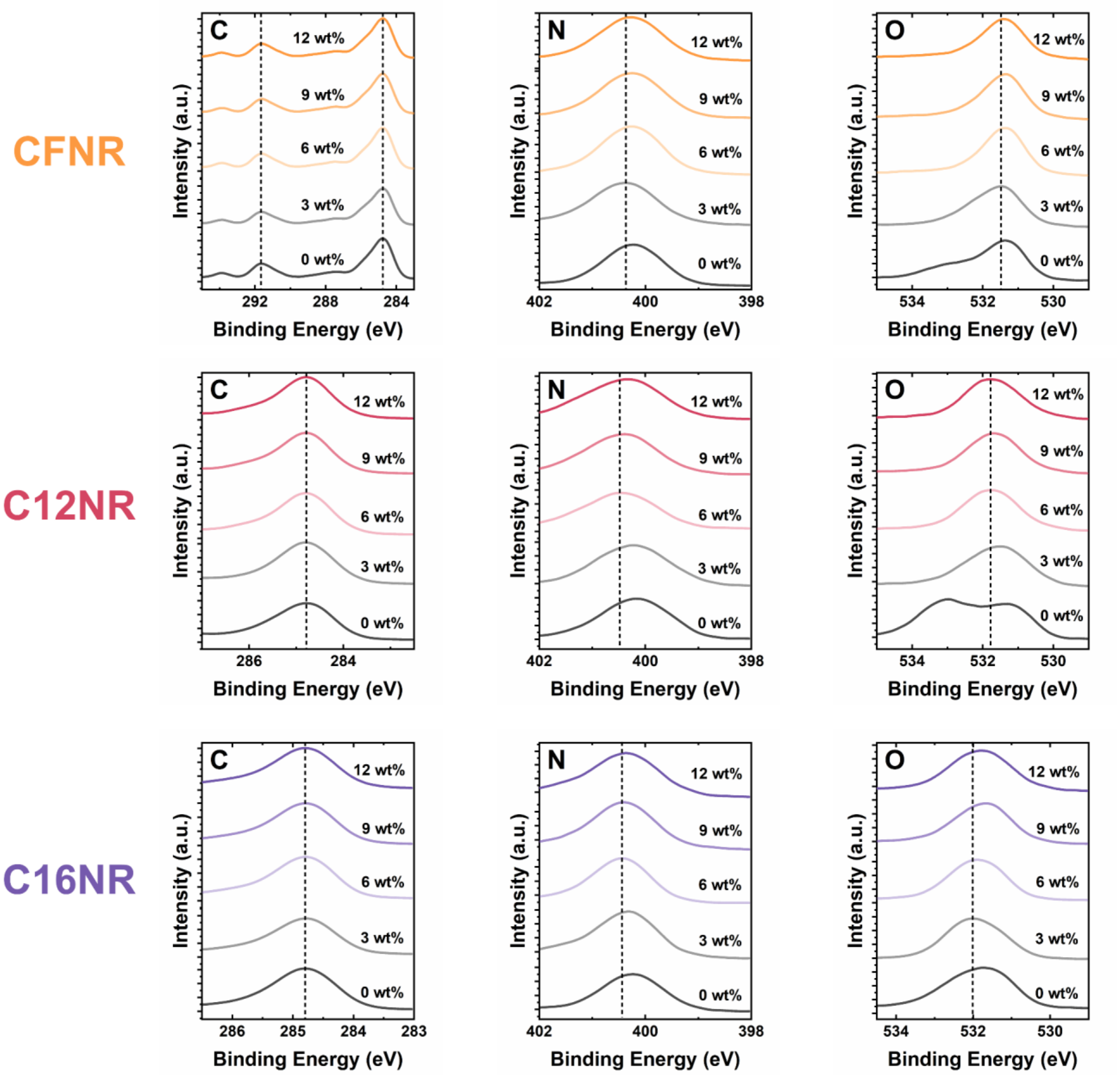

Figure S5. C 1S, O 1s and N 1s XPS spectra of **CFNR**, **C12NR** and **C16NR** thin film under different dopant concentration of N-DMBI.

6. AFM Images of Compounds

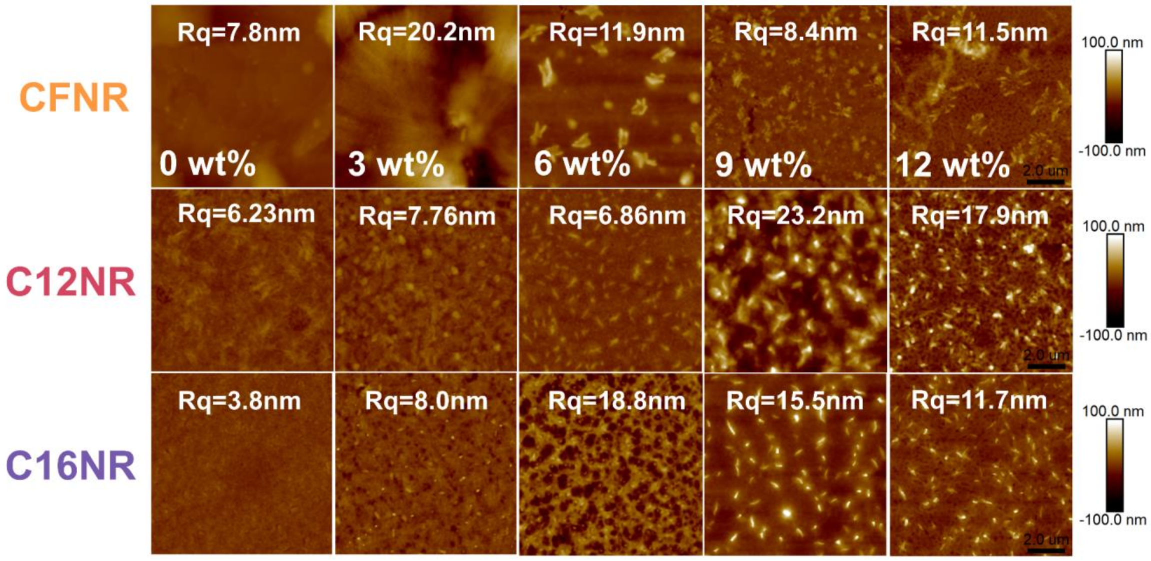

Figure S6. AFM images (10 × 10 μm) of semiconductor films of **CFNR**, **C12NR** and **C16NR** under different dopant concentration of N-DMBI.

7. The Details of the GIWAXs Study

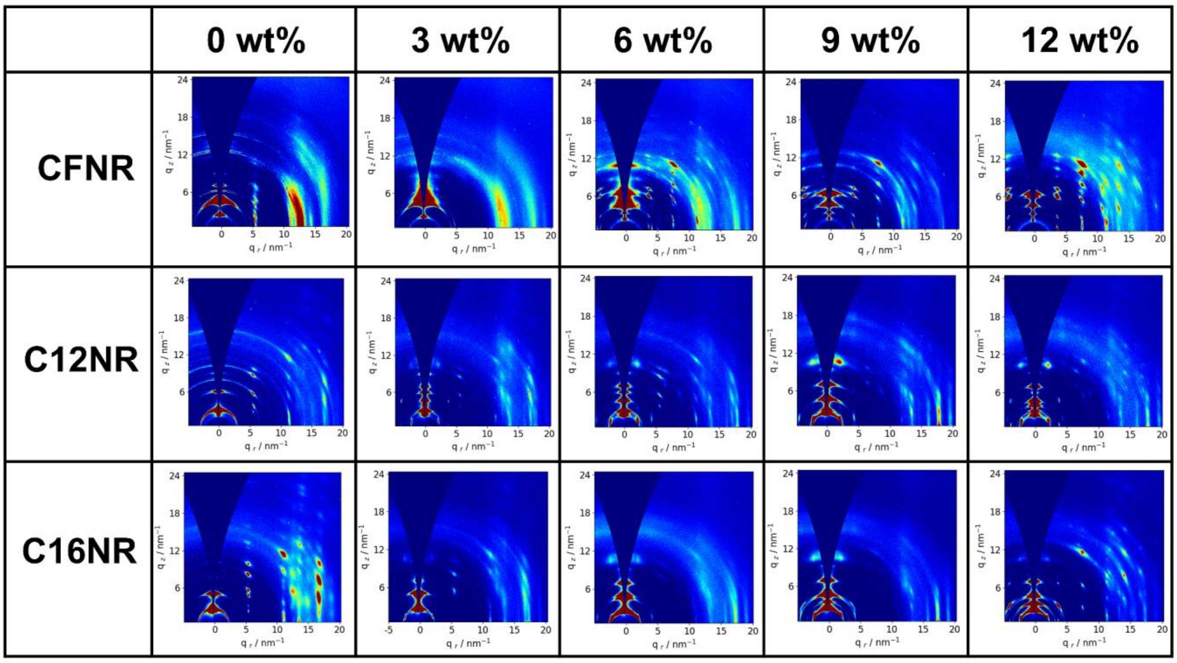

Figure S7. 2D-GIWAXs images of **CFNR**, **C12NR** and **C16NR** thin film under different dopant concentration of N-DMBI.

Table S1. Solid states packing parameters of **CFNR** in the out-of-plane (OOP) and in-plane (IP) direction.

	CFNR	0 wt%	3 wt%	6 wt%	9 wt%	12 wt%
**Lamellar packing (100)**	q (A^{-1})	0.206	0.191	0.196	0.195	0.195
	d-spacing (A)	30.49	32.88	32.04	32.20	32.21
**Lamellar packing (200)**	q (A^{-1})	0.419	0.409	0.418	0.420	0.420
	d-spacing (A)	15.00	15.35	15.02	14.95	14.95
**Lamellar packing (100) D**	q (A^{-1})	N/A	N/A	0.289	0.289	0.293
	d-spacing (A)			21.73	21.73	21.43
**Lamellar**	q (A^{-1})	N/A	N/A	0.603	0.611	0.611

**packing (200)** [D]	d-spacing (A)			10.41	10.28	10.28
**π-π stack (010) In plane**	q (A^{-1})	1.705	1.740	1.759	1.805	1.805
	d-spacing (A)	3.68	3.61	3.57	3.48	3.48

Table S2. Solid states packing parameters of **C12NR** in the out-of-plane (OOP) and in-plane (IP) direction.

	C12NR	0 wt%	3 wt%	6 wt%	9 wt%	12 wt%
**Lamellar packing (100)**	q (A^{-1})	0.268	0.264	0.265	0.262	0.262
	d-spacing (A)	23.43	23.79	23.70	23.97	23.97
**Lamellar packing (200)**	q (A^{-1})	0.565	0.556	0.562	N/A	N/A
	d-spacing (A)	11.12	11.29	11.27		
**Lamellar packing (100) D**	q (A^{-1})	N/A	0.202	0.202	0.203	0.197
	d-spacing (A)		31.09	31.09	30.94	31.88
**Lamellar packing (200) D**	q (A^{-1})	N/A	0.430	0.431	0.434	0.422
	d-spacing (A)		14.60	14.57	14.47	14.88
**Lamellar packing (300) D**	q (A^{-1})	N/A	0.654	0.660	0.668	0.649
	d-spacing (A)		9.6	9.5	9.4	9.7
**π-π stack (010) In plane**	q (A^{-1})	1.730	1.736	1.761	1.767	1.766
	d-spacing (A)	3.63	3.62	3.57	3.55	3.56

Table S3. Solid states packing parameters of **C16NR** in the out-of-plane (OOP) and in-plane (IP) direction.

	C16NR	0 wt%	3 wt%	6 wt%	9 wt%	12 wt%
**Lamellar packing (100)**	q (A^{-1})	0.231	0.223	0.220	0.220	N/A
	d-spacing (A)	27.19	28.16	28.55	28.55	
**Lamellar packing (200)**	q (A^{-1})	0.476	0.475	0.467	N/A	N/A
	d-spacing (A)	13.19	13.22	13.45		
**Lamellar packing (100) D**	q (A^{-1})	N/A	0.203	0.199	0.199	0.203
	d-spacing (A)		30.94	31.56	31.56	30.94
**Lamellar packing (200) D**	q (A^{-1})	N/A	0.433	0.427	0.430	0.434
	d-spacing (A)		14.50	14.71	14.60	14.47
**Lamellar packing (300) D**	q (A^{-1})	N/A	0.663	0.651	0.657	0.664
	d-spacing (A)		9.47	9.65	9.56	9.46
**π-π stack (010) In plane**	q (A^{-1})	1.722	1.741	1.740	1.744	1.745
	d-spacing (A)	3.65	3.61	3.61	3.60	3.60

8. Hall effect measurements

Table S4. Summary of the Hall voltage (V_H), Hall coefficient (R_H), carrier concentration (n) and mobility (μ) from Hall effect measurements on **CFNR**, **C12NR** and **C16NR** thin films with 6 wt% N-DMBI

	V_H (mV)	R_H (cm^3 C^{-1})	n (cm^{-3})	μ (cm^2 V^{-1} s^{-1})
CFNR		N/A		
C12NR	-0.19	-1.11×10^{-2}	5.63×10^{20}	0.002
C16NR		N/A		

The electrical conductivity of CFNR and C16NR is too low to measure the Hall signal.

9. Materials Synthesis and Characterization

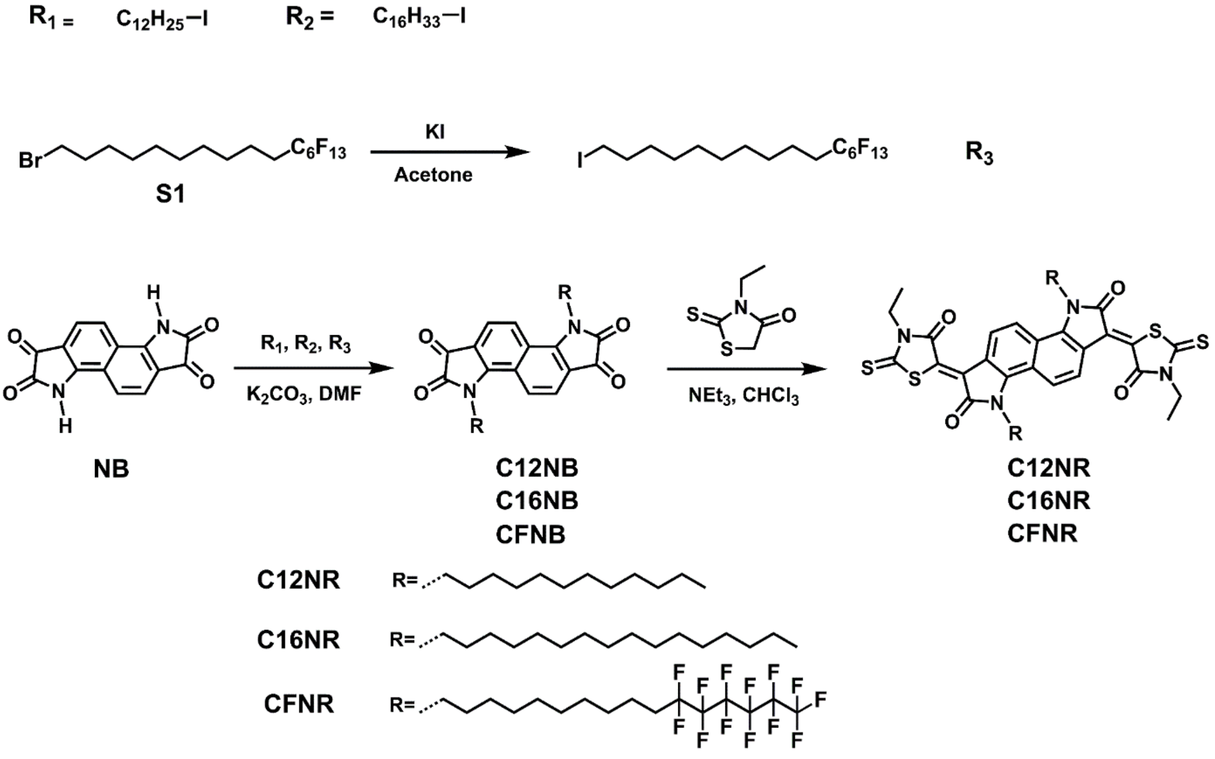

Scheme S1. Synthetic route of the NR derivatives. $\mathbf{R_1}$[1], $\mathbf{R_2}$[2], **S1**[3], **NB** [4] and **C12NB** [4] were synthesized according to the literature.

R_3

To a stirred solution of **S1** (10.0 g, 18.6 mmol, 1.0 equiv) in acetone (200 mL) was added KI (12.3 g, 74.3 mmol, 4.0 equiv). The resulting solution was stirred at 56 ^{o}C overnight. The solvent was removed under vacuum. The crude product was washed with hexane and filtered. The final product was obtained as slightly yellow liquid (9.8 g, 16.8 mmol, 90%). ^{1}H NMR

(400 MHz, $CDCl_3$, 300 K), δ (ppm): 3.21 (t, 2H), 2.07 (m, 2H), 1.85 (m, 2H), 1.62 (m, 2H), 1.28-1.50 (m, 12H).

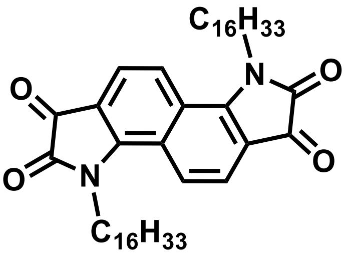

C16NB

To a suspension of **NB** (1.0 g, 3.76 mmol, 1.0 equi.) in anhydrous DMF (15.0 mL) was added freshly dried K_2CO_3 (2.1 g, 15.03 mmol, 4.0 equi.). The mixture was heated to 88 ^oC. After 0.5 h, 1-iodohexadecane (**R$_2$**) (4.0 g, 11.27 mmol, 3.0 equi.) was added in one portion. The reaction mixture was stirred at 88 ^oC for 1 h. The reaction mixture was cooled down to room temperature and poured over water and acidified with 1M HCl to pH of 7. The aqueous layer was extracted with DCM, washed with water, brine and dried over $MgSO_4$. The solvent was removed under vacuum. The crude product was purified by column chromatography (silica gel, DCM), washed with hexane and filtered. The final product was obtained as blue solid (600 mg, 0.84 mmol, 22%).

^{1}H NMR (600 MHz, $CDCl_3$, 300 K), δ (ppm): 7.98 (d, J = 6.60 Hz, 2H), 7.71 (d, J = 6.78 Hz, 2H), 4.25 (t, 4H), 1.84 (m, 4H), 1.23-1.48 (m, 52H), 0.90 (t, 6H). ^{13}C NMR (600 MHz, $CDCl_3$, 300 K), δ (ppm): 182.82, 159.04, 152.23, 127.30, 120.26, 119.62, 116.24, 43.53, 31.92, 29.68, 29.65, 29.51, 29.46, 29.35, 29.34, 29.17, 26.67, 22.69, 14.11. HRMS (MALDI-TOF): Calculated for $C_{46}H_{70}N_2O_4$: 714.5336, found $[M+H]^+$: 715.5408.

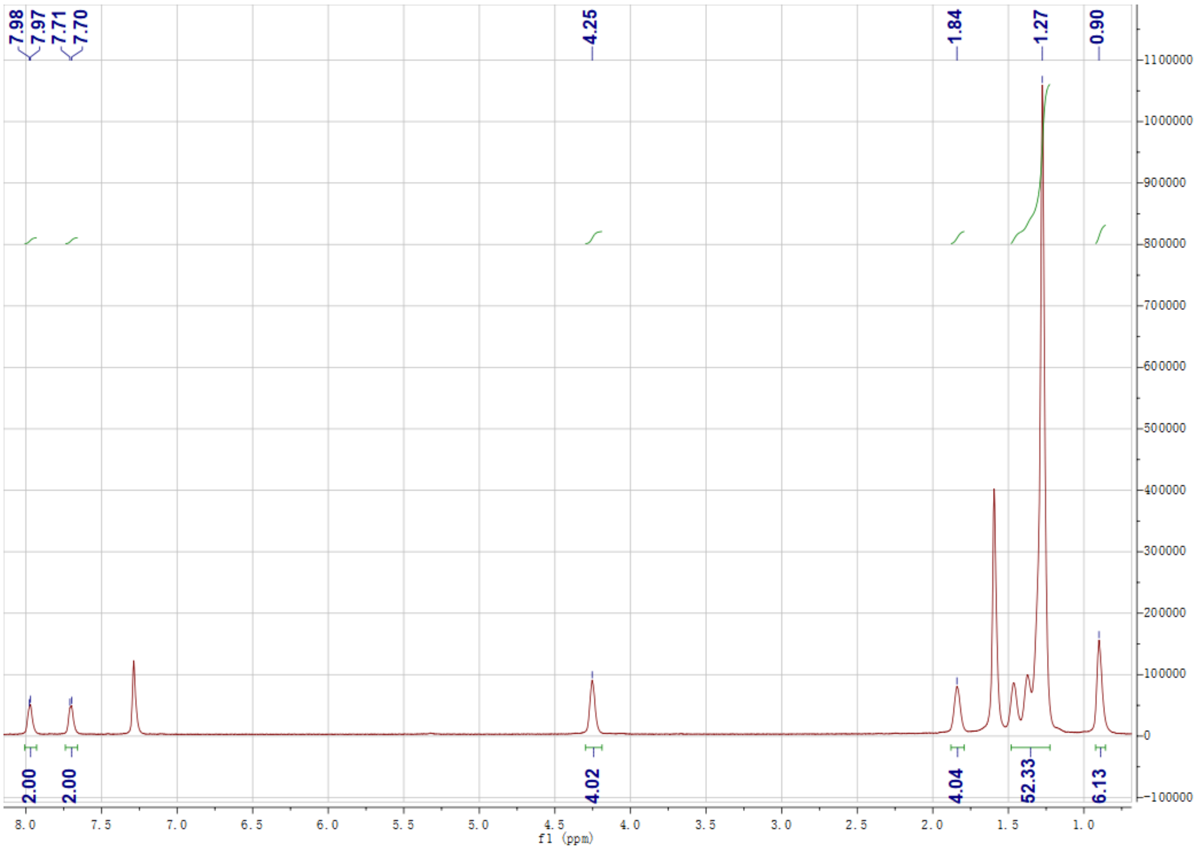

Figure S8. ^{1}H NMR of **C16NB** at 300 K.

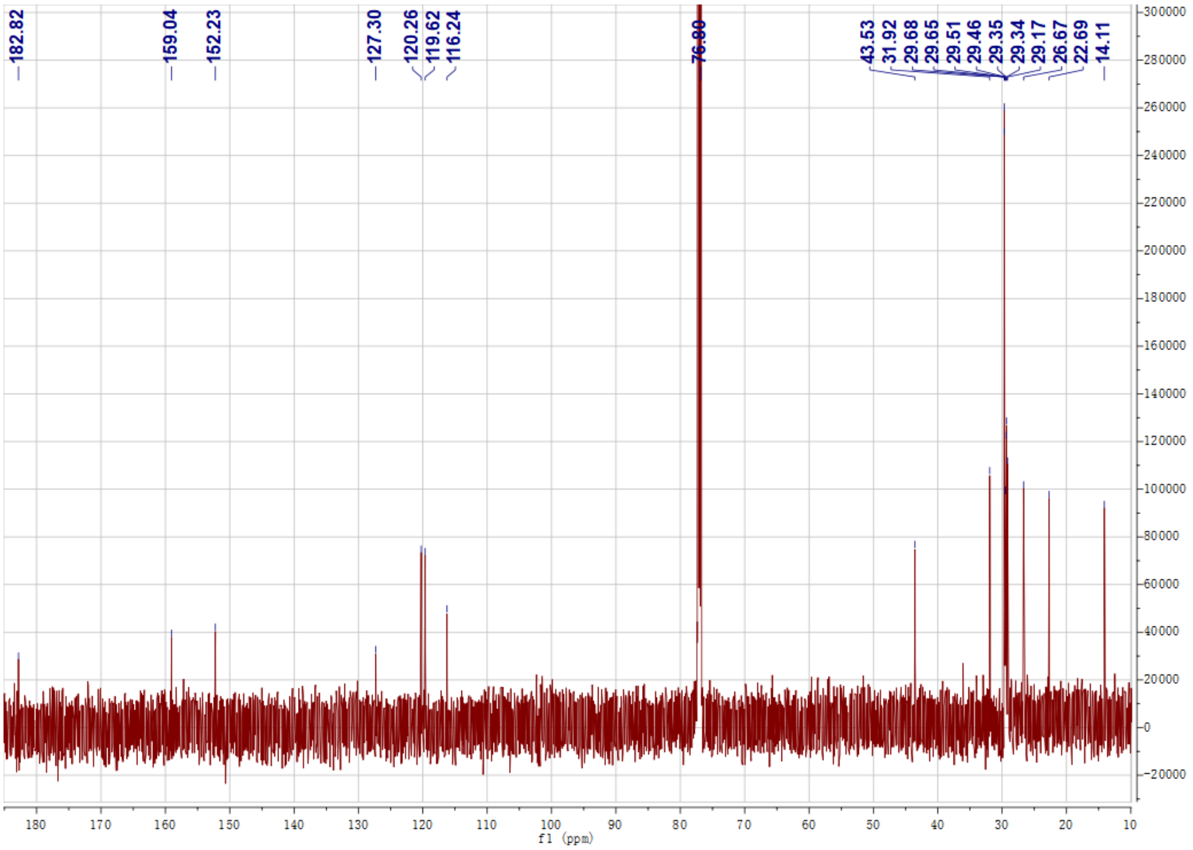

Figure S9. ^{13}C NMR of **C16NB** at 300 K.

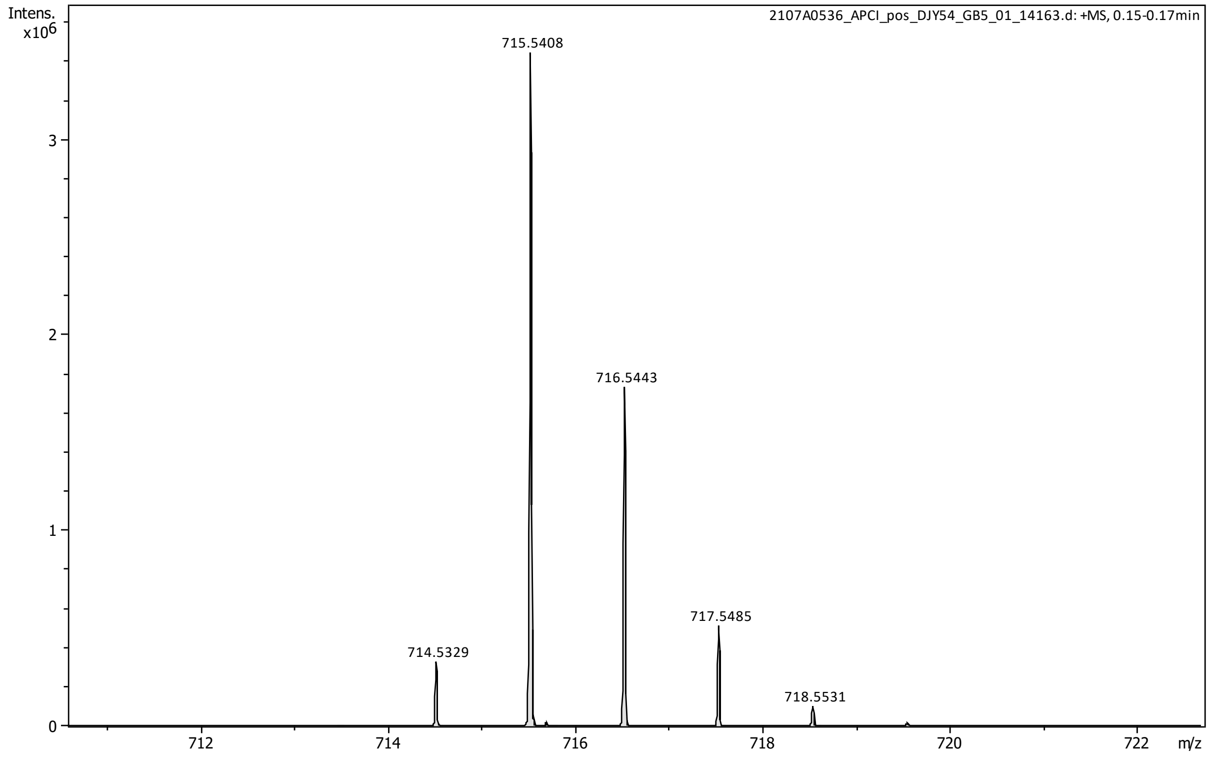

Figure S10. MALDI-TOF of **C16NB**.

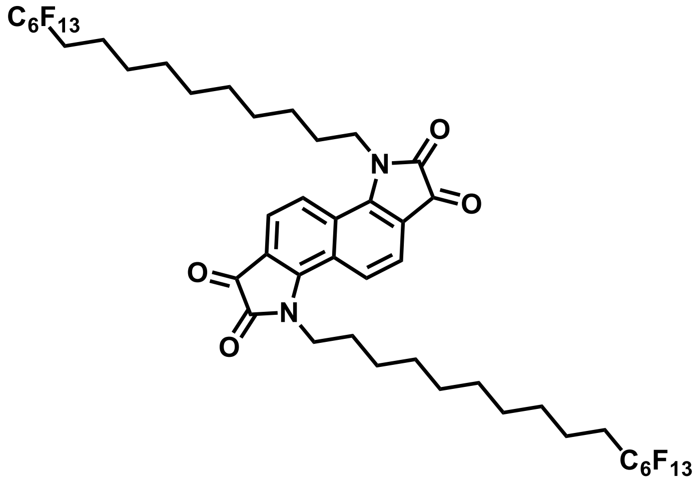

CFNB

CFNB was synthesized according to compound **C16NB**. The final product was obtained as blue solid (25%). ^{1}H NMR (600 MHz, $CDCl_3$, 300 K), δ (ppm): 7.98 (d, J = 8.64 Hz, 2H),

7.71 (d, J = 8.58 Hz, 2H), 4.26 (t, 4H), 2.06 (m, 4H), 1.84 (m, 4H), 1.26-1.51 (m, 28H). ^{13}C NMR (100 MHz, $CDCl_3$, 300 K), δ (ppm): 182.78, 159.03, 152.20, 127.29, 120.26, 119.60, 116.23, 43.47, 32.43, 31.01, 30.87, 30.72, 29.36, 29.34, 29.23, 29.16, 29.12, 29.05, 28.93, 28.80, 28.71, 26.65, 20.08. MALDI-TOF: Calculated for $C_{46}H_{44}F_{26}N_2O_4$: 1182.289, found: 1182.287.

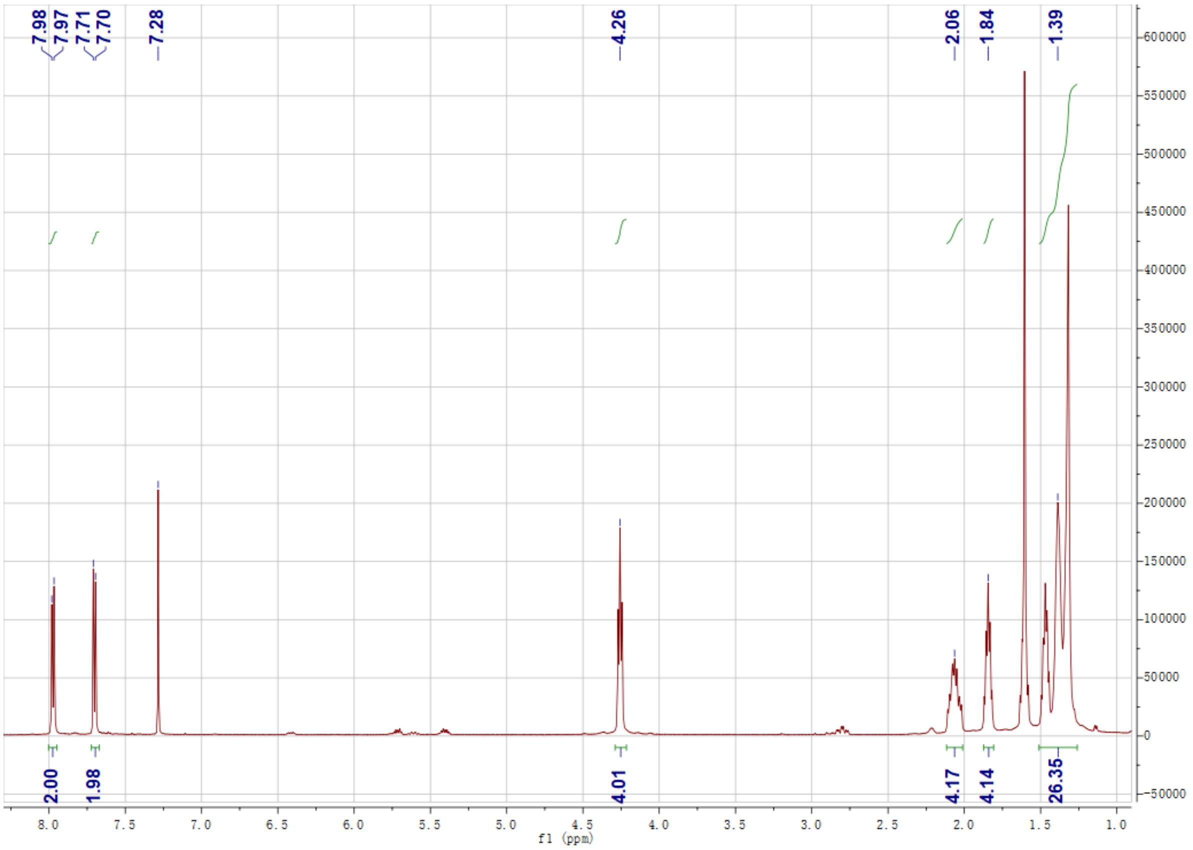

Figure S11. ^{1}H NMR of **CFNB** at 300 K.

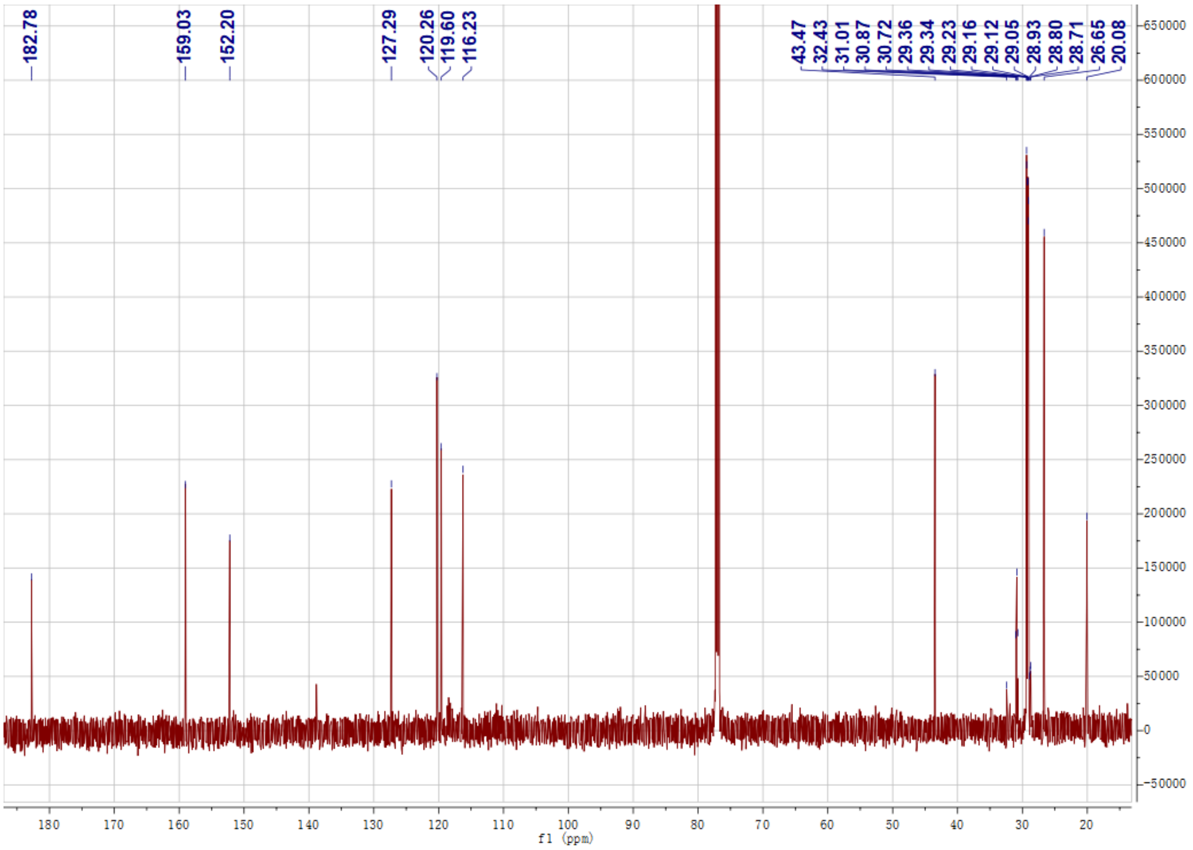

Figure S12. ^{13}C NMR of **CFNB** at 300 K.

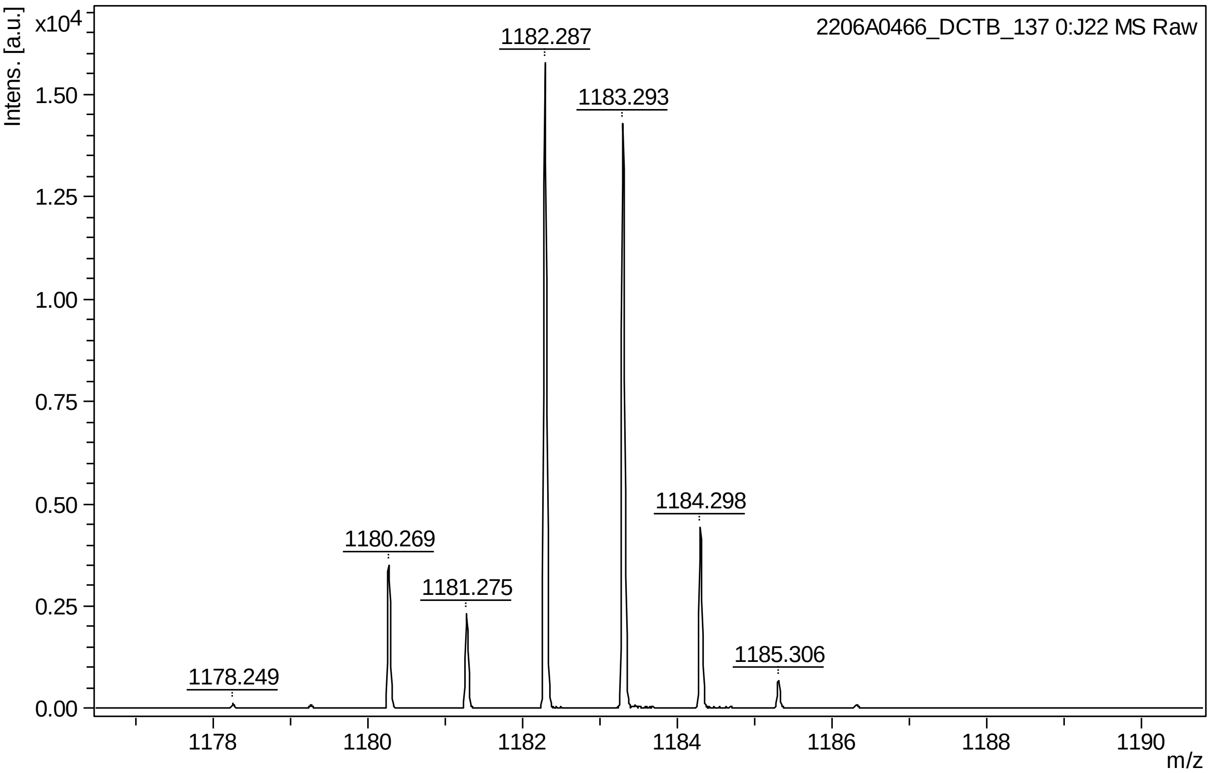

Figure S13. MALDI-TOF of **CFNB**.

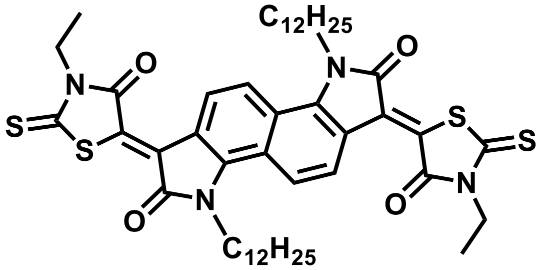

C12NR

In a 50 ml flame-dried Schlenk flask was added **C12NB** (50.0 mg, 0.083 mmol, 1.0 equi.) in anhydrous chloroform (10 mL), triethylamine (three drops), and 3-ethylrhodanine (33.5 mg, 0.208 mmol, 2.5 equi.) under nitrogen atmosphere. After that, the mixture was heated to reflux. The reaction mixture was stirred until the complete disappearance of the starting material was determined by thin layer chromatography. Then, the solvent was evaporated under vacuum, and the residue was purified by column chromatography (silica gel, DCM),

washed with methanol and filtered. The final product was obtained as green solid (54 mg, 0.061, 73%)

^{1}H NMR (500 MHz, TCE-D_2, 393 K), δ (ppm): 9.05 (d, J = 9.15 Hz, 2H), 7.88 (d, J = 9.25 Hz, 2H), 4.19-4.35 (m, 8H), 1.88 (m, 4H), 1.29-1.53 (m, 42H), 0.88 (t, J = 6.75 Hz, 6H). ^{13}C NMR (125 MHz, TCE-D_2, 393 K), δ (ppm): 196.36, 168.83, 166.91, 143.75, 134.26, 124.08, 123.24, 118.43, 117.42, 44.16, 39.67, 31.81, 29.52, 29.46, 29.41, 29.17, 26.80, 22.51, 13.83, 12.26. HRMS (MALDI-TOF): Calculated for $C_{48}H_{64}N_4O_4S_4$: 888.3810, found $[M+H]^+$: 889.3888.

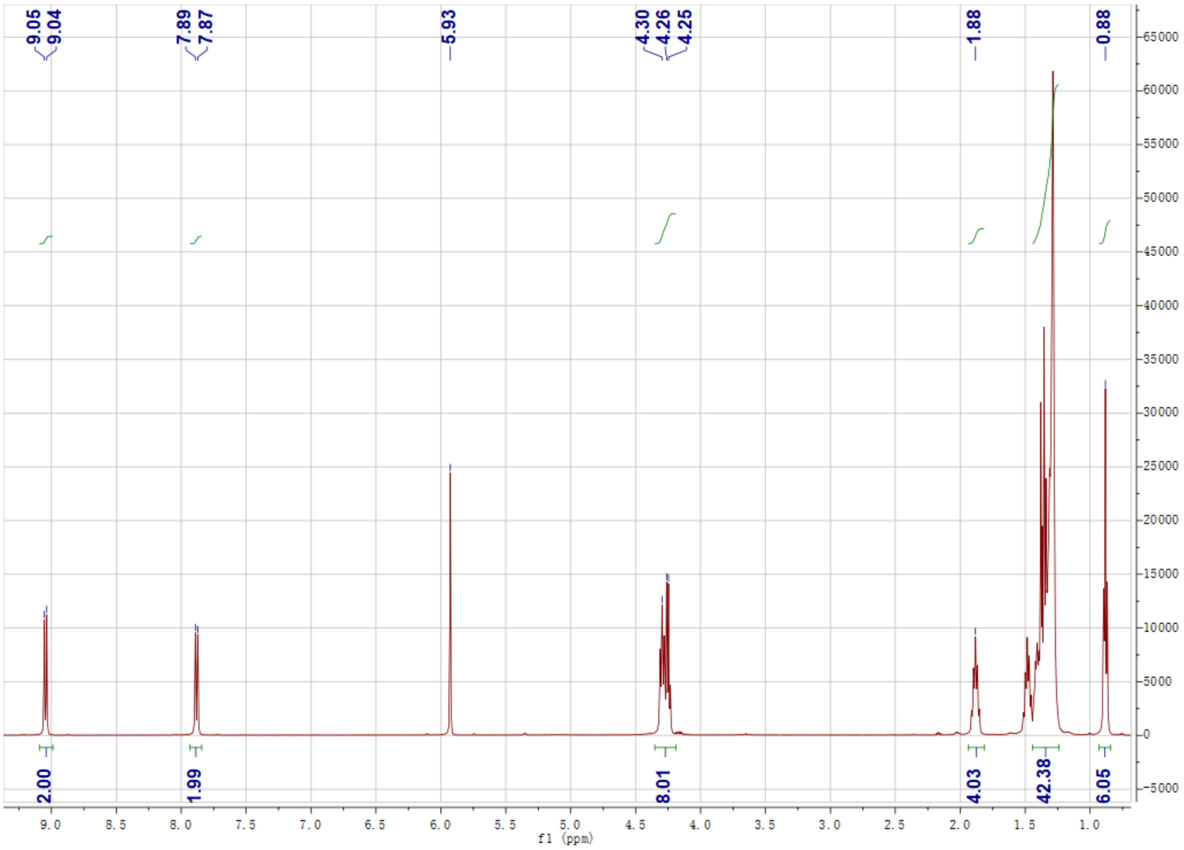

Figure S14. ^{1}H NMR of **C12NR** at 393 K.

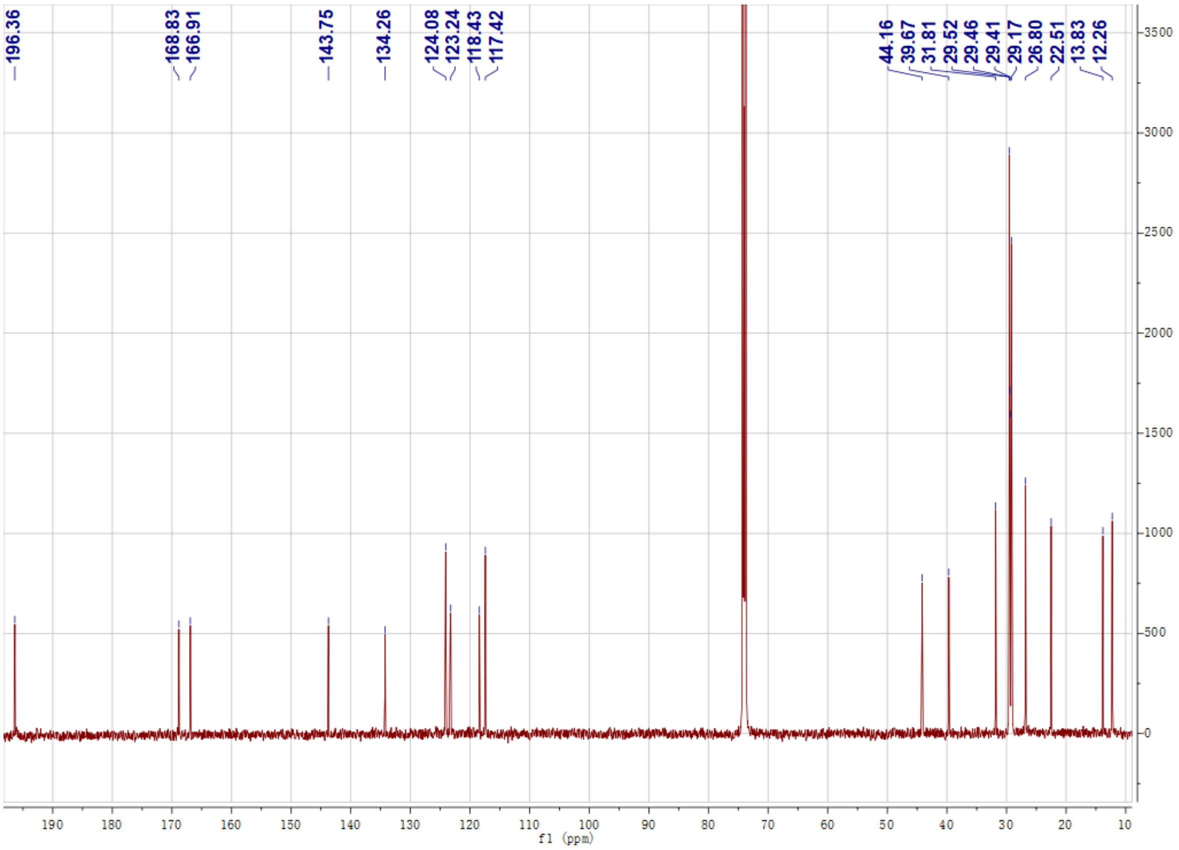

Figure S15. ^{13}C NMR of **C12NR** at 393 K.

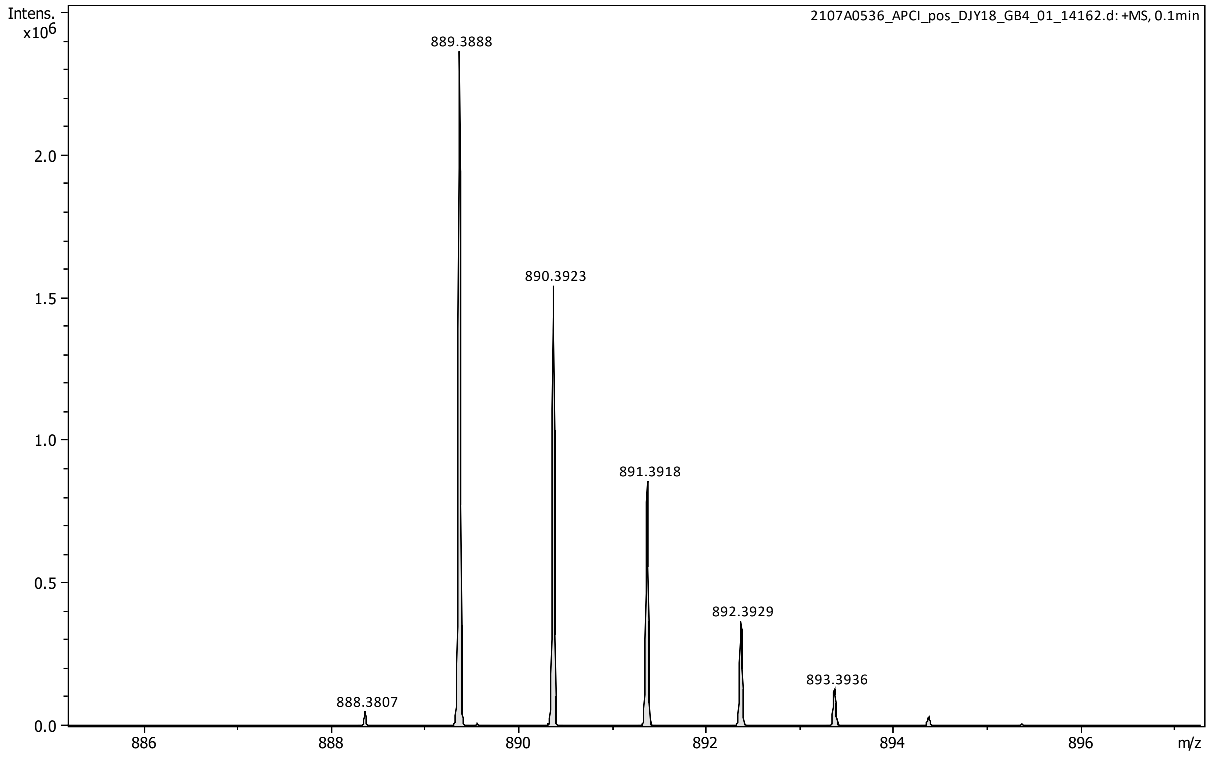

Figure S16. MALDI-TOF of **C12NR**.

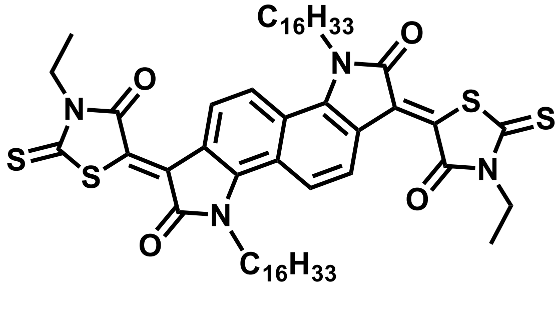

C16NR

C16NR was synthesized according to compound **C12NR**. The final product was obtained as green solid (77%). ^{1}H NMR (500 MHz, TCE-D$_2$, 393 K), δ (ppm): 9.06 (d, J = 9.15 Hz, 2H), 7.90 (d, J = 9.20 Hz, 2H), 4.22-4.35 (m, 8H), 1.88 (m, 4H), 1.24-1.40 (m, 58H), 0.88 (t, J = 6.75 Hz, 6H). ^{13}C NMR (125 MHz, TCE-D$_2$, 393 K), δ (ppm): 196.37, 168.85, 166.93,

143.77, 134.26, 124.11, 123.27, 118.46, 117.44, 44.16, 39.67, 31.82, 29.58, 29.54, 29.47, 29.42, 29.18, 26.80, 22.51, 13.83, 12.26. HRMS (MALDI-TOF): Calculated for $C_{56}H_{80}N_4O_4S_4$: 1000.5062, found $[M+H]^+$: 1001.5135.

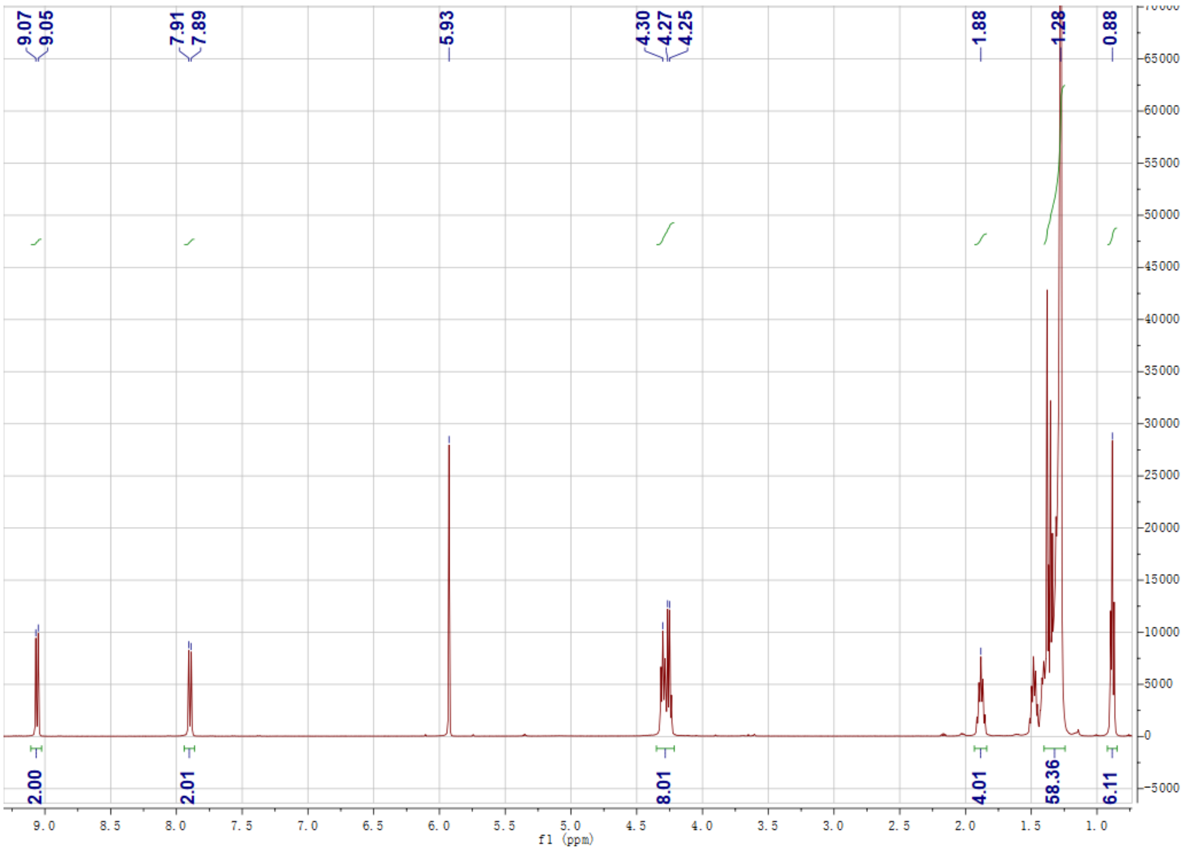

Figure S17. ^{1}H NMR of **C16NR** at 393 K.

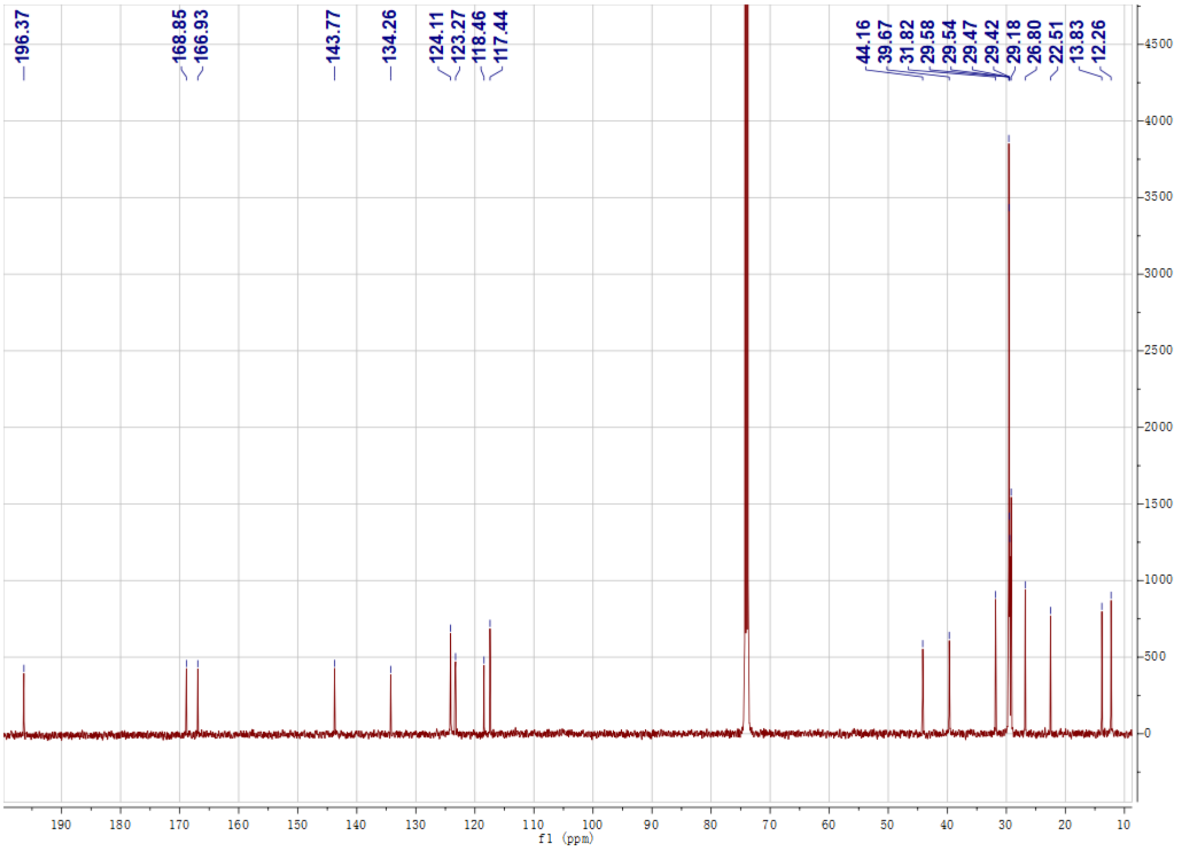

Figure S18. ^{13}C NMR of **C16NR** at 393 K.

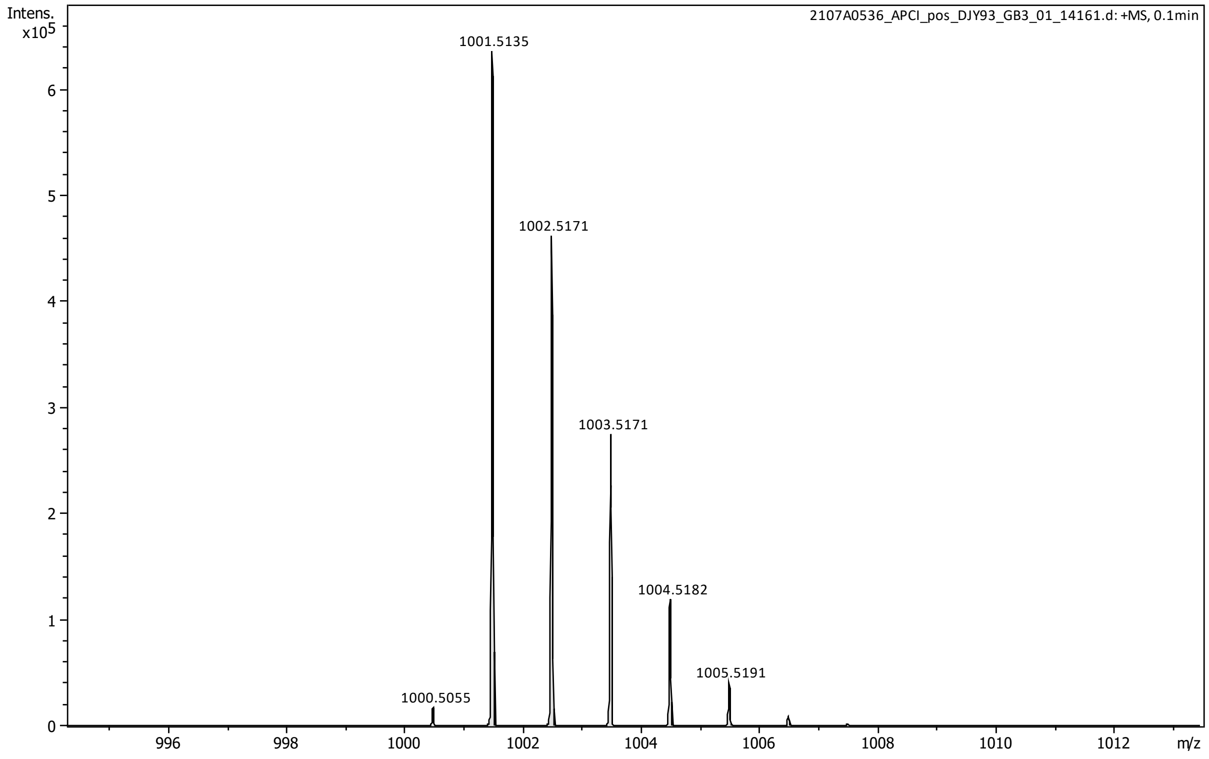

Figure S19. MALDI-TOF of **C16NR**.

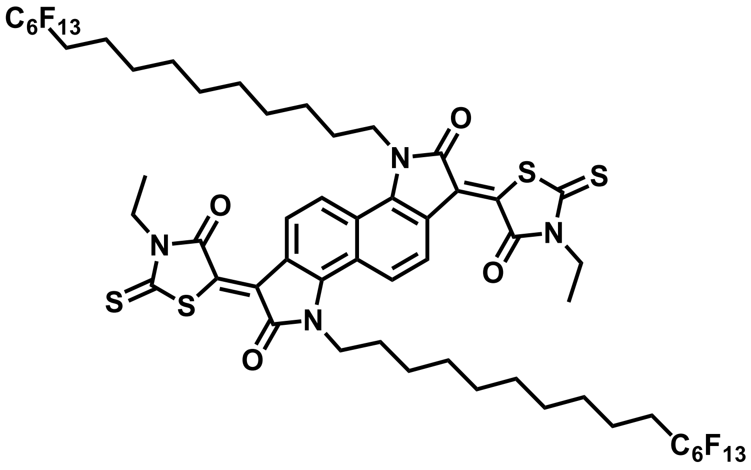

CFNR

CFNR was synthesized according to compound **C12NR**. The final product was obtained as green solid (80%). ^{1}H NMR (500 MHz, TCE-D_2, 393 K), δ (ppm): 9.08 (d, J = 9.20 Hz, 2H),

7.92 (d, J = 9.25 Hz, 2H), 4.20-4.37 (m, 8H), 2.06 (m, 4H), 1.89 (m, 4H), 1.60 (m, 4H), 1.49 (m, 4H), 1.31-1.40 (m, 26H). ^{13}C NMR (125 MHz, TCE-D_2, 393 K), δ (ppm): 196.33, 168.88, 166.97, 143.77, 134.34, 124.14, 123.30, 118.49, 117.43, 44.11, 39.65, 31.46, 31.28, 31.10, 29.53, 29.28, 29.15, 29.09, 29.06, 29.03, 26.76, 20.28, 12.22. HRMS (MALDI-TOF): Calculated for $C_{56}H_{54}F_{26}N_4O_4S_4$: 1468.2613, found $[M+H]^+$: 1469.2689.

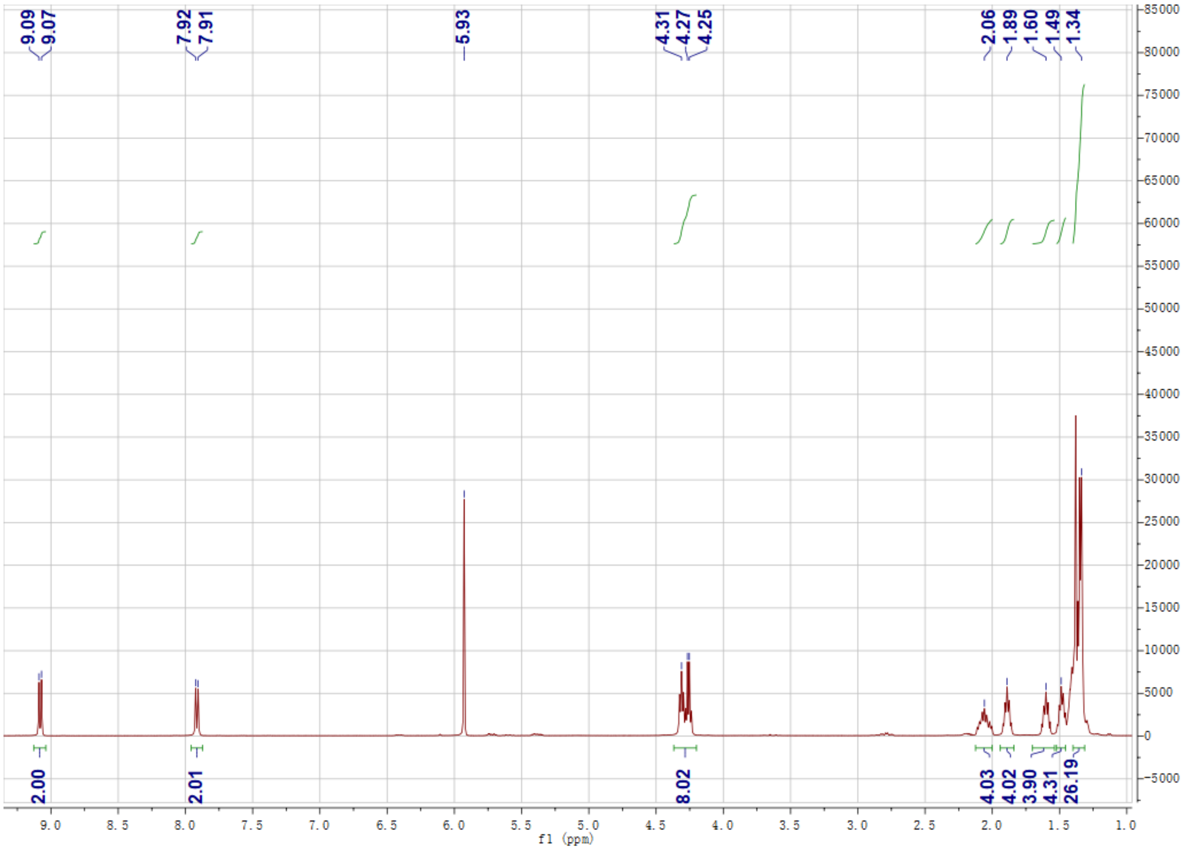

Figure S20. ^{1}H NMR of **CFNR** at 393 K.

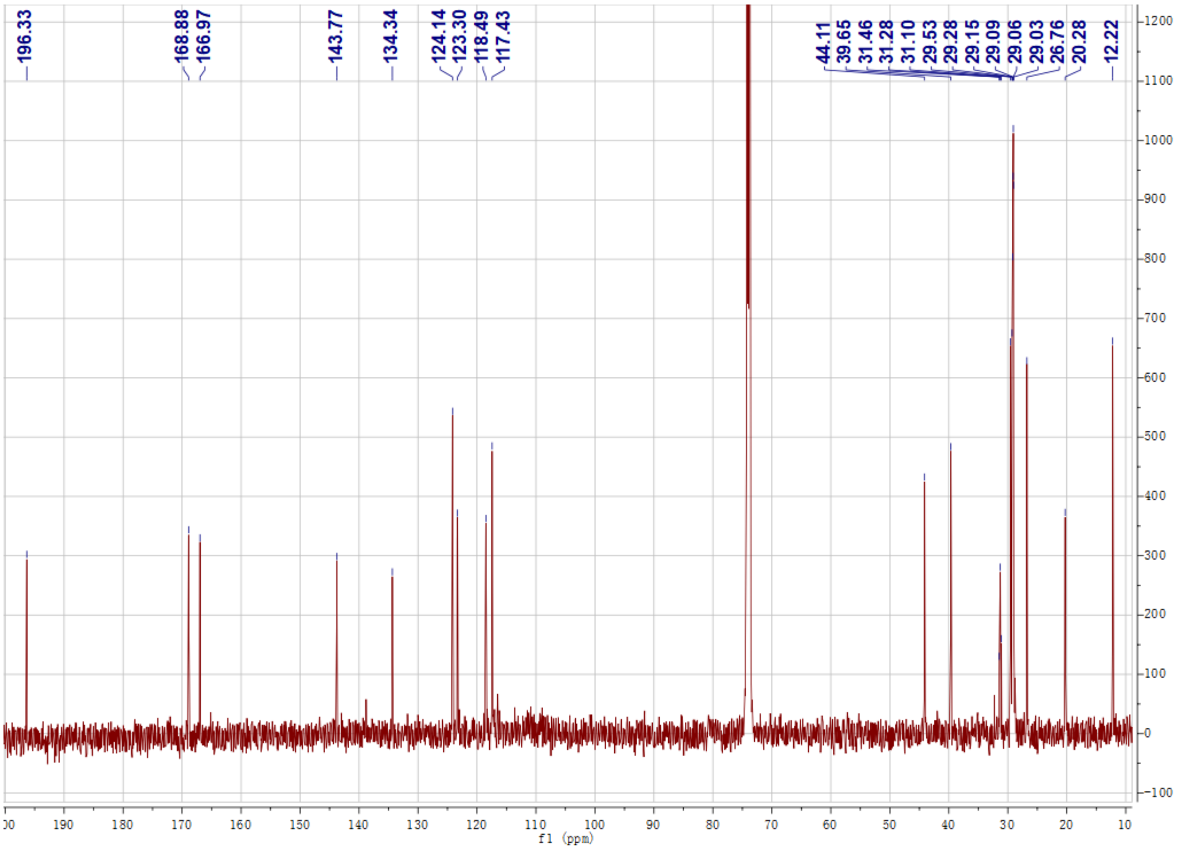

Figure S21. ^{13}C NMR of **CFNR** at 393 K.

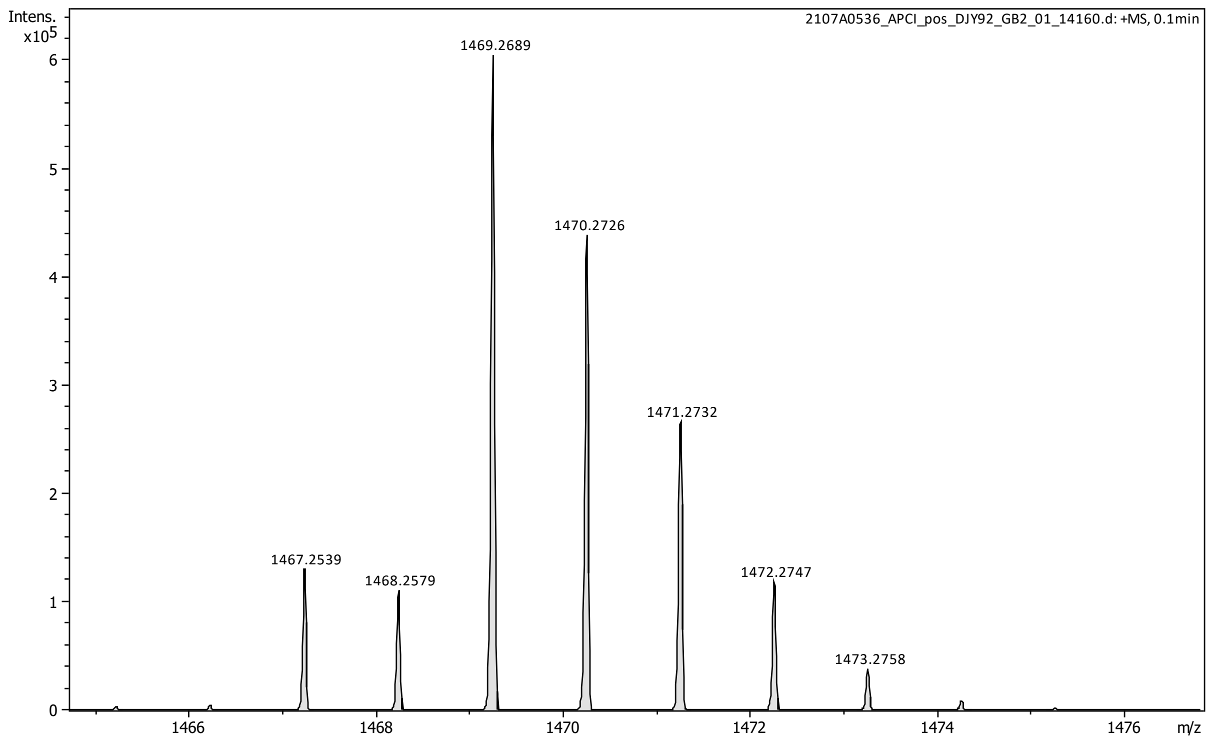

Figure S22. MALDI-TOF of **CFNR**.

10. References

[1] C. Depken, F. Kratzschmar, R. Rieger, K. Rode, A. Breder, *Angew. Chem. Int. Ed.* **2018**, 57, 2459-2463.

[2] D. Kalaitzakis, M. Triantafyllakis, I. Alexopoulou, M. Sofiadis, G. Vassilikogiannakis, *Angew. Chem. Int. Ed.* **2014**, 53, 13201-5.

[3] H. Miyajima, M. C. Z. Kasuya, K. Hatanaka, *Journal of Fluorine Chemistry* **2019**, 222-223, 24-30.

[4] A. Onwubiko, W. Yue, C. Jellett, M. Xiao, H. Y. Chen, M. K. Ravva, D. A. Hanifi, A. C. Knall, B. Purushothaman, M. Nikolka, J. C. Flores, A. Salleo, J. L. Bredas, H. Sirringhaus, P. Hayoz, I. McCulloch, *Nat. Commun.* **2018**, 9, 416.
